# Gonadotropin‐Inhibitory Hormone (GnIH) and Its Receptor GPR147 Mediates Acute Stress‐Induced Activation of the Locus Coeruleus‐SAM Axis

**DOI:** 10.1002/advs.202518996

**Published:** 2026-10-11

**Authors:** Jiani Zhang, Hongyu Zhu, Wenqi Wang, Bingqian Shen, Yuanyuan Xin, Xingxing Song, Xuanyi Lu, Lixuan Li, Siting Chen, Xiaochen Zhang, Jialing Zeng, Haodong Zheng, Jianlong Lao, Ke Peng, Yixian Wei, Jiaming Zheng, Chuanhuo Hu, Xiaoye Wang, Xun Li

**Affiliations:** ^1^ College of Animal Science and Technology Guangxi University Guangxi Key Laboratory of Animal Reproduction Breeding and Disease Control Guangxi Zhuang Autonomous Region Engineering Research Center of Veterinary Biologics Nanning People's Republic of China

**Keywords:** acute stress, anxiety‐like behavior, gonadotropin‐inhibitory hormone (GnIH), LC‐SAM axis, sympathetic neurotransmitter

## Abstract

Gonadotropin‐inhibitory hormone (GnIH, also known as RFamide‐related peptide‐3, RFRP‐3) is a hypothalamic neuropeptide that regulates vertebrate reproduction via its receptor GPR147. Although GnIH is known to mediate stress‐induced reproductive suppression, its role in broader stress responses remains unclear. This study investigated the fuction of GnIH in acute stress and evaluated the mechanisms by which GnIH regulates norepinephrine (NE) release and activates the locus coeruleus‐sympathetic‐adrenal medulla (LC‐SAM) axis in mice. The results show that GnIH is co‐localized and co‐stored with NE in sympathetic nerve terminals and that it is associated with heightened LC‐SAM axis activity induced by acute stressors. Central or peripheral administration of GnIH potentiated acute stress responses and activated the LC‐SAM axis, accompanied by increased anxiety‐like behaviors. Conversely, genetic ablation of GnIH or GPR147 attenuated acute stress‐induced anxiety‐like behaviors and LC‐SAM axis activation. Mechanistically, both central and peripheral GnIH enhanced NE synthesis, transport and release. These findings indicate that GnIH mimics or augments endogenous NE signaling to activated the LC‐SAM axis during acute stress. Pharmacological modulation of the GnIH/GPR147 system may represent a therapeutic approach for the inhibiting maladaptive stress responses and stress‐related pathologies and infertility.

## Abstract 2

The GnIH(RFRP‐3)/GPR147 system mediates acute stress‐induced activation of the LC‐SAM axis and anxiety‐like behaviors. GnIH mimics or augments endogenous norepinephrine signaling to activate this axis during acute stress. Pharmacological targeting of this system may offer a therapeutic strategy for maladaptive stress responses, stress‐related pathologies, and infertility.

## 1 Introduction

Stress serves as a phylogenetically conserved adaptive mechanism that enables humans and animals to mount coordinated responses to environmental threats or perceived internal homeostatic imbalances [1]. Nevertheless, chronic or maladaptive stress represents a principal etiological factor in the pathogenesis of psychiatric disorders (e.g., anxiety and depression) and systemic pathologies (including cardiovascular diseases, metabolic dysregulation, and oncogenesis) [2, 3]. This dual‐edged nature of stress is conceptualized through allostatic load‐the paradoxical phenomenon whereby evolutionarily conserved stress‐response mechanisms transition from protective to pathogenic states under conditions of sustained activation or regulatory failure [4]. Critically, the molecular determinants governing the transition from adaptive allostasis to maladaptive allostatic overload remain incompletely elucidated. Although the border between acute and chronic stress is diffuse, accumulating evidence supports that pathophysiological change caused by acute stress are often the basis of biological effects caused by chronic stress and may determine the direction of chronic stress‐induced response [5]. Consequently, delineating the neurobiological and neuroendocrine mechanisms underlying acute stress responses has emerged as a strategic approach for identifying dynamic biomarkers of allostatic load dynamics [6].

The neurophysiological architecture of stress adaptation manifests through multisystem integration, orchestrated by interconnected brain regions (e.g., amygdala, hypothalamus, and locus coeruleus (LC)) via bidirectional neurotransmission and neuroendocrine signaling [7]. This integrative process mobilizes both the sympatho‐adreno‐medullary (SAM) axis and hypothalamic‐pituitary‐adrenal (HPA) axis, culminating in the systemic release of NE and glucocorticoids (GC) [8]. Notably, noradrenergic neurons exhibit functional pleiotropy, co‐releasing neurotransmitter (e.g., neuropeptide Y (NPY), ghrelin and galanin) alongside NE, thereby unveiling a previously underestimated complexity in sympathetic neurotransmission during stress adaptation [9, 10]. These discoveries not only refine our mechanistic understanding of sympathetic nervous system (SNS) regulation but also nominate novel druggable targets for mitigating allostatic overload through pharmacological intervention.

Emerging evidence implicates gonadotropin‐inhibitory hormone (GnIH; also known as RFamide‐related peptide‐3, RFRP‐3), a hypothalamic neuropeptide acting via its receptor GPR147, in the intersection of stress responses and reproductive dysfunction [11, 12]. Elevated GnIH expression under diverse stress paradigms suggests its evolutionary role in prioritizing survival over reproduction via suppression of gonadal axis activity and energy conservation [13]. Intriguingly, GnIH neurons within the paraventricular nucleus (PVN) express α2A adrenergic receptors and receive direct noradrenergic projections in quail [14]. In rats, studies have characterized the anatomical proximity of GnIH neurons to noradrenergic projections in the brainstem, and intracerebroventricular (i.c.v) administion of GnIH activates noradrenergic populations in the locus coeruleus (LC) and nucleus tractus solitarius (NTS) [15, 16]. While direct physiological evidence for noradrenergic inputs and α2A receptor expression in rodent GnIH neurons is still lacking, the combined avian, rodent anatomical, and functional data establish a strong basis for noradrenergic regulation of the GnIH across species.

Building upon this framework, the present study aims to elucidates the functional interplay between the GnIH/GPR147 system and acute stress responses, with particular emphasis on its mechanistic regulation of LC‐SAM axis activation and NE release. We first provided anatomical relationship between GnIH/GPR147 system and LC‐SAM axis, and then profiled the synthesis and secretion of GnIH under diverse acute stress paradigms. Subsequent pharmacological and genetic perturbation experiments (i.e., GnIH administration, and GnIH or GPR147 knockout models) dissected the causal roles of GnIH in acute stress physiology, LC‐SAM axis mobilization, and anxiety‐like behaviors. To unravel the neuromodulatory mechanisms underlying GnIH regulated NE release, we further characterized the spatial coordination (co‐localization), biosynthesis, transport, and exocytosis of NE within LC‐SAM circuitry using adrenal explants and sympathetic nerve‐ablated mice receiving i.c.v GnIH treatments.

## 2 Materials and Methods

### 2.1 Animals

#### 2.1.1 Source and Breeding

Wide‐type C57BL/6 J mice (8‐9 weeks old, body weight 20–25 g, male) were purchased from Changsha Tianqin Biotechnology Company (Changsha, China). All animals were group‐housed on 12 h light‐dark cycle (lights on from 9:00 AM to 9:00 PM) at 25°C, with free access to food and water. Mice were allowed to acclimate to the housing conditions for one week prior to experimentation. All animal experimental procedures were performed in accordance with the guidelines of the regional Animal Ethics Committee and were pre‐approved by the Guangxi University Ethical Committee.

Mutant mice (GnIH^−/−^, GnIH^−/+^, GPR147^−/−^, GPR147^−/+^) were generated by Caygen Biological Company (Guangzhou, China) using the CRISPR/Cas9 system in the C57BL/6J mouse strain. To create a GnIH knockout mouse, Exons 1 to 3 were targeted, encompassing a 567 bp coding sequence within the region. Specifically, gRNA1 (GATATTCTATACACGCTAGCTGG) targets exon 1, and gRNA2 (CTTCTCCAGACCTAGTGAACAGG) targets exon 3.

#### 2.1.2 Genotyping of GnIH Mutant Mice

Genomic DNA was extracted from mouse tail tips, and two sequential PCR reactions were performed. The primer sequences used for the first PCR were: GnIH‐F (5′‐CAT TTG CCA AAT TAG ACC CTT AGG G‐3′) and GnIH‐R (5′‐AAA TGC AAC CCA GGG TTG ATG TC‐3′). Based on sequence integrity, the expected sizes of the mutant and wild‐type alleles from the first PCR product were 588 bp and 4964 bp, respectively. The primer sequences used for the second PCR were: GnIH‐F (5′‐CAT TTG CCA AAT TAG ACC CTT AGG G‐3′), GnIH‐R (5′‐AAA TGC AAC CCA GGG TTG ATG TC‐3′), and GnIH‐He/Wt‐R (5′‐CAG AAG GTG TTT GAT GTT AAG AAG C‐3′). Based on sequence integrity, the expected sizes of the homozygous mutant, heterozygous mutant, and wild‐type alleles from the second PCR product were 588 bp, 588 + 759 bp, and 759 bp, respectively. Mouse genotypes were determined by combining the band patterns from the two PCR products.

#### 2.1.3 Genotyping of GPR147 Mutant Mice

Genomic DNA was extracted from mouse tail tips, and two sequential PCR reactions were performed. The primer sequences used for the first PCR were: GPR147‐F (5′‐GTG GAC AGT AAT AAG TGG GCT TAG GGT‐3′) and GPR147‐R (5′‐AGC TAA ACA ACA GTC TCC TGC ATG‐3′). Based on sequence integrity, the expected sizes of the mutant and wild‐type alleles from the first PCR product were 810 bp and 10,756 bp, respectively. The primer sequences used for the second PCR were: GPR147‐F (5′‐GTG GAC AGT AAT AAG TGG GCT TAG GGT‐3′), GPR147‐R (5′‐AGC TAA ACA ACA GTC TCC TGC ATG‐3′), and GPR147‐He/Wt‐R (5′‐GGG AGC ACA AGA GTC AGG ACC AA‐3′). Based on sequence integrity, the expected sizes of the homozygous mutant, heterozygous mutant, and wild‐type alleles from the second PCR product were 810 bp, 910 + 653 bp, and 653 bp, respectively. Mouse genotypes were determined by combining the band patterns from the two PCR products.

### 2.2 Drugs Administration

GnIH was synthesized by the Beijing Genomics Institute, Beijing, China. Polypeptide sequence is Phe‐Pro‐Ser‐Leu‐Pro‐Gln‐Arg‐Phe‐NH_2_ and its purity is >98%.

For ablation of sympathetic nerves, 6‐hydroxydopamine hydrobromide (6‐OHDA, Cat. 162957, Merck Co. Ltd., Germany) solution was prepared freshly by dissolving 6‐OHDA in 0.1% ascorbic acid in 0.9% sterile NaCl. Mice received intraperitoneal injections of 6‐OHDA at a dose of 100 mg/kg body weight once daily for five consecutive days [17].

For GnIH intraperitoneal (i.p) treatments, mice (WT, n = 10 per group) received acute i.p injections of GnIH (20 µg/100 µL of GnIH dissolved in a 0.9% saline solution). The dose of GnIH for i.p injection was based on our previous studies [18]. Control mice were injected with an equivalent volume of vehicle.

For GnIH i.c.v treatments, mice (WT and sympathetic nerves ablation pretreated mice, n = 10 per group) were anaesthetised with isoflurane in oxygen and positioned in the stereotaxic apparatus (71001‐D, RWD Co. Ltd., China). Microinjector was lowered to the lateral ventricle at 1 mm lateral to the midline, 0.8 mm posterior to the bregma, and 2.3 mm inferior to the dura matter [19]. Mice received ventricular injections 1 µL of either GnIH (50 ng) or artificial cerebrospinal fluid (aCSF) over 2 min [20]. The dose of GnIH for i.c.v injection refer to previous studies [21].

For GnIH intra‐LC treatments, mice (WT, n = 10 per group) were anesthetized with isoflurane in oxygen and placed in a stereotaxic apparatus (71001‐D, RWD Co., Ltd., China). Using bregma as the reference point, the intra‐LC injection coordinates were set as anteroposterior (AP) = −5.4 mm, mediolateral (ML) = ±1.0 mm, and dorsoventral (DV) = −3.8 mm. The dose of GnIH for intra‐LC injection was the same as that used for i.c.v. injection. Each mouse received a single intra‐LC injection of 0.3 µL of either GnIH or aCSF over 2 min.

After 15 min GnIH administration, all groups of mice were sacrificed by decapitation under 3% isoflurane (Cat. R510‐22‐10, RWD Co. Ltd., China). The serum, a part of brainstem and adrenal gland from each mouse were collected and kept at −80°C, while another part of brainstem and adrenal gland was fixed in neutral‐buffered formalin for histology analysis.

### 2.3 Acute Stress Paradigm

Acute restraint, heat and immunological stress procedures were performed as previously described [22, 23, 24]. In brief, for acute restraint stress, mice were kept individually in a 50 mL plastic centrifuge tube with ventilation holes to restricted their movement for 60 min. For heat stress, mice were placed into ten cages with one mouse in each cage and moved to a temperature‐controlled chamber (LRH‐250A, Taihong Medical Equipment Co. Ltd., China) maintained at 40°C ± 1°C and 60 ± 10% humidity for 1 h. During the thermal stress process, the diet and water intake of mice were not restricted. For immunological stress, lipopolysaccharide (LPS, Cat. l2880, Merck Co. Ltd., Germany) was intraperitoneally injected at a dose of 0.5 mg/kg dissolved in saline vehicle. After stress procedures, mice were anesthetized with 3% isoflurane. The serum, a part of brainstem and adrenal gland from each mouse were collected and kept at −80°C, while another part of brainstem and adrenal gland was fixed in neutral‐buffered formalin for morphological analysis.

### 2.4 Behavioral Tests

The GnIH‐treated and control mice as well as WT, GnIH or GPR147‐KO mice subjected to acute restraint stress for 45 min, were used to investigate anxiety‐related emotional and defensive behaviors immediately after stress exposure. Behavioral assessments included the open‐field test (OFT), elevated plus‐maze test (EPM), and light‐dark box (LDB) test, which were conducted as previously described [25, 26, 27] with minor modifications.

#### 2.4.1 Open‐Field Test (OFT)

The open‐field apparatus consisted of an open‐topped plastic box (50 × 50 × 40 cm) with black plastic walls and a white floor. The white floor was divided into 25 squares (10 × 10 cm): 16 squares along the walls designated as the peripheral areas and 9 central squares designated as the central area. Mice were allowed to acclimate to the testing environment for 60 min before the experiment. Each test was initiated by placing a mouse on the central platform, after which it was allowed to freely explore the arena for 5 min. Locomotor activity and path traces were recorded and analyzed using VisuTrack software (XR‐VT, Shanghai Xinruan Information Technology Co., Ltd, China). The arena was carefully cleaned with 70% ethanol and dried with a clean cloth after each test. The anxiety index was calculated by the following formula [28]. Anxiety index = 1‐ [(time in center/5 min) + (distance travelled in center/total distance travelled)]/2.

#### 2.4.2 Elevated Plus‐Maze (EPM) Test

The elevated plus‐maze was positioned 55 cm above the floor and consisted of two open arms (5 × 35 × 15 cm) and two closed arms (5 × 35 × 15 cm) made of black plastic. Mice were allowed to acclimate to the testing site for 60 min. At the start of the test, each mouse was placed in the central platform (5 × 5 cm) facing one of the open arms and was allowed to freely explore both open and closed arms for 5 min. Behavior was continuously recorded by a video camera positioned above the maze. VisuTrack software was used to capture video and analyze ethological parameters. The maze was cleaned with 70% ethanol and dried with a clean cloth after each test. The anxiety index was calculated as follows: Anxiety index = 1‐ [(time in open arm/5 min) + (open arm entries/total entries)]/2.

#### 2.4.3 Light‐Dark Box (LDB) Test

Each mouse was placed in the center of a light compartment (27 × 18 × 18 cm, white surfaces) connected to a dark compartment (18 × 18 × 18 cm, black surfaces) by a square aperture. Mice were allowed to freely explore the entire apparatus for 10 min. Both compartments were individually equipped with a micro‐camera. The apparatus was carefully cleaned with 70% ethanol and dried with a clean cloth after each test.

### 2.5 ELISA

Concentrations of circulating stress hormones, including corticosterone (CORT; Cat. MM‐0061M1, Jiangsu Meimian Industrial Co., Ltd., China), norepinephrine (NE; Cat. MM‐0627M1, Jiangsu Meimian Industrial Co., Ltd., China), epinephrine (EPI; Cat. MM‐0351M1, Jiangsu Meimian Industrial Co., Ltd., China), and GnIH (Cat. EDL202412024, Shanghai Kanglang Biological Technology Co., Ltd., China), were measured using commercial enzyme‐linked immunosorbent assay (ELISA) kits according to the manufacturer's protocols. Absorbance was measured at 450 nm using an automatic microplate reader (Bio‐Rad 680, Bio‐Rad, USA). To assess blood‐brain barrier (BBB) permeability of GnIH, we measured GnIH concentrations in serum and CSF following i.p. injection. All samples were run in triplicate. Assay sensitivities were as follows: 2 µg/L for CORT, 7.5 pg/mL for NE, 0.1 ng/mL for EPI, and 20pg/mL for GnIH. The intra‐assay and inter‐assay coefficients of variation (CVs) for each hormone were determined at low, medium, and high concentrations of reference samples. For CORT, the intra‐assay CVs were 4.32%, 8.09%, and 2.06%, and the inter‐assay CVs were 7.68%, 5.26%, and 3.88%, respectively. For EPI, the intra‐assay CVs were 4.59%, 11.13%, and 9.93%, and the inter‐assay CVs were 4.81%, 8.68%, and 12.29%, respectively. For NE, the intra‐assay CVs were 6.05%, 6.09%, and 9.41%, and the inter‐assay CVs were 7.13%, 6.88%, and 8.17%, respectively. For GnIH, the intra‐assay CVs were 2.78%, 11.49%, and 7.56%, and the inter‐assay CVs were 9.69%, 17.9%, and 14.11%, respectively.

### 2.6 Morphological Analysis

#### 2.6.1 Immunohistochemistry and Immunofluorescence

Fixed brainstem, thoracolumbar spinal cord, and adrenal tissues were rinsed overnight under running water. The samples were then dehydrated through a graded ethanol series, embedded in paraffin wax, and sectioned at 5 µm thickness using a microtome (RM2235, Leica Biosystems Inc., Germany). Antibody specificity for immunohistochemistry and immunofluorescence was verified as described in our previously study [29]. For immunohistochemistry and immunofluorescence, tissue sections were incubated with primary antibodies at the appropriate dilution. The following primary antibodies were used: Tyrosine Hydroxylase (TH; Cat. sc‐25269, Santa Cruz, 1:100 dilution; immunogen: human TH amino acids 1–196; species reactivity: mouse; label dopaminergic cells); GPR147 (Cat. Orb157323, Biorbyt, 1:250 dilution; immunogen: keyhole limpet hemocyanin (KLH)‐conjugated synthetic peptide derived from human GPR147/NPFF1R; species reactivity: mouse); GnIH (raised in our laboratory, 1:500 dilution; immunogen: KLH‐conjugated synthetic peptide derived from mouse NPVF/RFRP amino acids); choline O‐acetyltransferase (ChAT; Cat. AMAB91130, Sigma, 1:1000 dilution; immunogen: human ChAT; species reactivity: mouse); neuropeptide Y (NPY; Cat. YP‐Ab‐12776, UpingBio, 1:100 dilution; immunogen: synthetic peptide derived from human NPY amino acids 45–94; species reactivity: mouse); and phenylethanolamine N‐methyltransferase (PNMT; Cat. sc‐393995, Santa Cruz, 1:100 dilution; immunogen: the internal region of human PNMT, amino acids 164–212; species reactivity: mouse; label adrenergic cells). Immunohistochemical analyses were performed using streptavidin‐biotin‐peroxidase complex (SABC) kits (Cat. SA1027, Boster Biological Technology Co. Ltd., China). Representative photomicrographs were acquired using a light microscope (BX53, Olympus Optical Co. Ltd., Japan). For immunofluorescence analysis, after primary antibody incubation, sections were incubated with Cy3‐conjugated goat anti‐mouse IgG (Cat. AS008, ABclonal, 1:200 dilution), Fluor 594‐conjugated AffiniPure donkey anti‐rabbit IgG (Cat. BA1147, Boster, 1:200 dilution), or FITC‐conjugated goat anti‐rabbit IgG (Cat. AS001, ABclonal, 1:200 dilution). Nuclei were counterstained with 4′,6‐diamidino‐2‐phenylindole (DAPI; Cat. AR1176, Beijing Solarbio Technology Co., Ltd., China). Fluorescence images were captured using a confocal laser confocal microscopy (FV3000, Olympus Optical Co. Ltd., Japan). For immunofluorescence analysis, after primary antibody incubation, the sections were incubated with Cy3‐conjugated goat anti‐ mouse IgG (Cat. AS008, ABclonal, 1:200 dilution) or Fluoro 594 Conjugated AffiniPure Donkey Anti‐Rabbit IgG (Cat. BA1147, Boster, 1:200 dilution), and FITC conjugated goat anti‐rabbit IgG secondary antibodies (Cat. AS001, ABclonal, 1:200 dilution). 4’,6‐Diamidino‐2 phenylindole (Cat. AR1176, Beijing Solarbio Technology Co. Ltd., China) was used to counterstain the nuclei, and the microscopic images were recorded by laser confocal microscopy (FV3000, Olympus Optical Co. Ltd., Japan).

#### 2.6.2 Dual‐Label Immuno‐Electron Microscopy

Dual‐label immuno‐electron microscopy (immuno‐EM) was performed as described in Renno's study [30].  Briefly, fresh brainstem and adrenal gland specimens were fixed and sectioned at 70–80 nm (Leica UC7, Leica, Germany). The grids were then incubated overnight at 4°C with primary antibodies: anti‐GnIH (1:500 dilution) and anti‐tyrosine hydroxylase (1:100 dilution). Following primary antibody incubation, the sections were incubated at room temperature for 20 min first, then transferred to a 37°C oven for 1 h, and finally returned to room temperature for rewarming for 0.5 h with secondary antibodies: goat anti‐rabbit IgG conjugated to 12 nm colloidal gold particles (Cat. 111‐205‐144, 1:25 dilution, Jackson, for GnIH detection) and goat anti‐mouse IgG conjugated to 4 nm colloidal gold particles (Cat. 115‐185‐146, 1:25 dilution, Jackson, for TH detection). Observations were made using a transmission electron microscope (HT7800, HITACHI, Japan), and images were captured for analysis.

### 2.7 Gene Expression

Relative real‐time RT‐PCR was performed as described in our previous study [31, 32, 33]. In brief, the total RNA was extracted using TRIzol reagent (Cat. R411‐01, Vazyme, Nanjing, China) for the brainstem and adrenal gland from mice. The first strand cDNA was synthesized from the total RNA using a Reverse Transcription Kit (Cat. R211‐02, Vazyme, Nanjing, China). Amplification reactions were conducted in triplicate using gene‐specific primers designed from the clone sequences shown in Table 1. The PCR products in each cycle were monitored using a fluorescence quantitative PCR instrument (BFQP‐96, Hangzhou Bigfish Bio‐tech Co., Ltd, China). The quantitative RT‐PCR was performed using the 2^−∆∆CT^ method with glyceraldehyde‐3‐phosphate dehydrogenase (GAPDH) as the internal control for the normalization.

**TABLE 1:** Primer sequences for quantitative real‐time PCR.

Genes	Full name	Genebank No.	Primer sequences (5’‐3’)	Annealing/°C
*GnIH*	Gonadotropin‐inhibitory hormone	NM_021892.2	F: TGATGCCTCATTTTCACAGCA R: CTGCGGGGCTTCTTTTCTC	60
*GPR147*	G‐protein coupled receptor 147	NM_001177511.2	F: CCACAACCCTCGTGGACAAC R: CCTGTACCAAGCCGCTCAT	61
*TH*	Tyrosine hydroxylase	NM_009377.2	F: TCTCCTTGAGGGGTACAAAACC R: ACCTCGAAGCGCACAAAGT	61
*PNMT*	Phenylethanolamine N‐methyltransferase	NM_008890.3	F: CGGGCTTTGCATCACATCAC R: GGACCTCGTAACCACCAAGG	62
*Dbh*	Dopamine Beta‐Hydroxylase	NM_138942.3	F: GGACCCCGAAGGGATTTTAGA R: CCATCTCTCCTCGATCTGACA	60
*Ddc*	dopa decarboxylase	NM_001190448.1	F: TAGCTGACTATCTGGATGGCAT R: GTCCTCGTATGTTTCTGGCTC	60
*NET*	Norepinephrine transporter	NM_009209.3	F: ATCATCGCCTGGTCACTCTAC R: GTTTGGGGTCCGTACAGTTGG	62
*VMAT2*	Vesicular monoamine transporter type 2	NM_172523.3	F: ATGCTGCTCACCGTCGTAG R: GGCAGTCTGGATTTCCGTAGT	61
*Syntaxin 1A*	Syntaxin 1A	NM_001359179.1	F: AAGATTGCCGAAAACGTGGAG R: TGCTCAATGCTCTTTAGCTTGG	60
*synaptophysin*	synaptophysin	NM_009305.2	F: AGACATGGACGTGGTGAATCA R: ACTCTCCGTCTTGTTGGCAC	62
*SNAP25*	Synaptosomal‐associated protein 25 kDa	NM_001291056.2	F: ATCCGCAGGGTAACAAATGATG R: CGGAGGTTTCCGATGATGC	60
*Ddit4*	DNA‐damage‐inducible transcript 4‌	NM_029083.2	F: CAAGGCAAGAGCTGCCATAG R: CCGGTACTTAGCGTCAGGG	61
*Irs2*	insulin receptor substrate 2	NM_001081212.2	F: ACCGACTTGGTCAGCGAAG R: CACGAGCCCGTAGTTGTCAT	62
*Dusp1*	Dual Specificity Phosphatase 1	NM_013642.3	F: TGTTGTTGGATTGTCGCTCCT R: TTGGGCACGATATGCTCCAG	62
*Sik1*	Salt‐Inducible Kinase 1	NM_010831.3	F: TCATGTCGGAGTTCAGTGCG R: ACCTGCGTTTTGGTGACTCG	62
*GAPDH*	glyceraldehyde‐3‐phosphate dehydrogenase	NM_001289726.2	F: GGGAAGCTCACTGGCATGG R: CTTCTTGATGTCATCATACTTGGCAG	60

### 2.8 Western Blot Analysis

Western blotting was conducted as described in our previous study [34, 35]. In brief, the brainstem and adrenal gland of mice were lysed in RIPA buffer (Cat.89901, Thermo Fisher Scientific Co., Ltd., America). The following primary antibodies were used: β‐tubulin (Cat. 66240‐1‐Ig, proteintech, 1:2000 dilution; immunogen: recombinant human Tubulin‐beta protein; species reactivity: mouse), TH (Cat. sc‐25269, Santa Cruz, 1:100 dilution; immunogen: human TH amino acids 1–196; species reactivity: mouse), norepinephrine transporter (NET) (Cat. 680447, Zenbio, 1:1000 dilution; immunogen: a synthetic peptide of human NET; species reactivity: mouse), synaptosomal‐associated protein, 25 kDa (SNAP25) (Cat. R27294, Zenbio, 1:1000 dilution; immunogen: human SNAP25; species reactivity: mouse), Syntaxin 1A (Cat. R389318, Zenbio, 1:1000 dilution; immunogen: human STX1A; species reactivity: mouse) and Synaptophysin (Cat. R380890, Zenbio, 1:1000 dilution; immunogen: human Synaptophysin amino acids 10–310; species reactivity: mouse). After incubation with primary antibodies, the membranes were then incubated with horseradish peroxidase (HRP)‐conjugated goat anti‐rabbit IgG secondary antibody (Cat. 7074P2, 1:20,000 dilution, Cell Signaling Technology) or anti‐mouse IgG secondary antibody (Cat. 7076P2, 1:20,000 dilution, Cell Signaling Technology). The densitometric quantification was performed with Image J (Version 1.53, NIH, Bethesda, MD, USA) using β‐tubulin as the internal control for the normalization.

### 2.9 LC‐MS/MS Analysis

The serum from GnIH or vehicle i.p treated mice were collected and measured 23 major serum neurotransmitter metabolites (Table 2) using targeted neurotransmitters metabolomics. It was conducted by Nomi Metabolism Detection Center (Suzhou PANOMIX Biomedical Tech Co., Ltd., China) as described in previous study [36]. All neurotransmitter reference standards were weighed, and standards at different concentrations were prepared using the corresponding solvents. The quantitative ranges and standard curves for the various neurotransmitter analytes (based on the quantification of 100 µL analysis of serum) are listed in Table 2. The proteins to be tested were precipitated from serum samples, evaporated to dryness in a centrifugal vacuum concentrator, and analyzed by mass spectrometry using an AB 4000 triple quadrupole mass spectrometer (waters acquity UPLC) and the 6460 triple quadrupole liquid chromatography‐mass spectrometry system from Suzhou PANOMIX Biomedical Tech Co., LTD. Multiple reaction monitoring (MRM) was used for scanning. Data were collected and processed using ab SCIEX Analyst software version 1.6.3.

**TABLE 2:** Targeted metabolomic profiling.

NO.	Abbreviation	Name	CAS	Linear equation of a standard curve	Correlation coefficient (r)	Range of linearity (ng/ml)
1	GABA	4‐Aminobutyric acid	56‐12‐2	y = 0.01687 x + 0.00429	0.99308	0.2‐200
2	HisA	Histamine	51‐45‐6	y = 0.00799 x + 0.01296	0.99315	0.5‐500
3	PA	Picolinic acid	98‐98‐6	y = 0.02503 x + 0.01381	0.99346	0.5‐250
4	TyrA	Tyramine	51‐67‐2	y = 0.04510 x + 0.03098	0.99357	0.4‐200
5	Ach	Acetylcholine	60‐31‐1	y = 0.03784 x + 0.01399	0.99929	1‐1000
6	Gln	L‐Glutamine	56‐85‐9	y = 0.04687 x + 0.00707	0.99959	0.5‐500
7	Glu	L‐Glutamic acid	56‐86‐0	y = 0.11892 x + −0.00866	0.99117	0.25‐125
8	DA	Dopamine	62‐31‐7	y = 0.02805 x + 0.00576	0.99482	0.5‐500
9	His	L‐Histidine	71‐00‐1	y = 0.06740 x + 0.00452	0.99597	0.2‐200
10	TrpA	Tryptamine	61‐54‐1	y = 0.15677 x + 0.00304	0.99598	0.1‐100
11	NE	Noradrenaline	55‐27‐6	y = 0.04396 x + 0.00173	0.99184	0.5‐500
12	5‐HT	Serotonin	153‐98‐0	y = 0.12753 x + 0.00475	0.99599	0.1‐100
13	Tyr	L‐Tyrosine	60‐18‐4	y = 0.00171 x + 0.00344	0.99460	2‐1000
14	E	Adrenaline	329‐63‐5	y = 0.04412 x + 2.36384e‐5	0.99805	0.1‐100
15	KynA	Kynurenic acid	492‐27‐3	y = 0.03796 x + 0.00287	0.99261	0.25‐250
16	5‐HIAA	5‐Hydroxyindole‐3‐acetic acid	54‐16‐0	y = 0.45144 x + 0.01167	0.99846	0.1‐100
17	DOPA	Levodopa	59‐92‐7	y = 0.07816 x + −0.00498	0.99703	0.5‐500
18	Trp	L‐Tryptophan	73‐22‐3	y = 0.04750 x + 1.46933e‐4	0.99409	1‐1000
19	XA	Xanthurenic acid	59‐00‐7	y = 0.00140 x + 0.00132	0.99597	2‐2000
20	Kyn	DL‐Kynurenine	343‐65‐7	y = 0.00767 x + 0.00117	0.99828	1‐1000
21	VMA	Vanillymandelic Acid	55‐10‐7	y = 0.00242 x + 0.00200	0.99822	5‐5000
22	5‐HTP	5‐Hydroxytryptophan	56‐69‐9	y = 0.09077 x + −0.00312	0.99511	0.2‐200
23	MT	Melatonine	73‐31‐4	y = 0.65653 x + 0.00405	0.99264	0.1‐100

### 2.10 RNA‐Sequencing

Total RNA samples were isolated from adrenal glands upon control and GnIH i.p treatment groups using TRIzol reagent (Cat. 15596026CN, Invitrogen, America) according to the manufacturer's instruction. RNA quality was determined by 5300 Bioanalyzer (Agilent, America) and quantified using the ND‐2000 (NanoDrop Technologies, America). RNA‐seq transcriptome library was prepared following TruSeqTM RNA sample preparation Kit (Cat. RS‐122‐2001, Illumina, America) using 1 µg of total RNA. The raw paired end reads were trimmed, and quality controlled by SeqPrep and FASTP with default parameters. Then clean reads were separately aligned to reference genome with orientation mode using HISAT2 software. The mapped reads of each sample were assembled by StringTie in a reference‐based approach.

RNA‐sequencing (RNA‐seq) was performed by Majorbio using the Illumina NovaSeq 6000 platform. To identify differentially expressed genes (DEGs) between the control and GnIH treatment groups, transcript expression levels were calculated using the DESeq2 method, and gene abundances were quantified with RSEM. The threshold for DEGs was set at a false discovery rate (FDR) < 0.05 and |log_2_ fold change| ≥ 1. RNA‐seq analyses, including Kyoto Encyclopedia of Genes and Genomes (KEGG) pathway enrichment, Gene Ontology (GO) annotation, Gene Set Enrichment Analysis (GSEA), and protein‐protein interaction (PPI) network topology analysis of stress‐related DEGs, were performed using the free online platform of the Majorbio Cloud Platform (www.majorbio.com). All the sequencing data have been submitted to the China National Center for Bioinformation.

### 2.11 Adrenal Explants Cultured In Vitro

Adrenal explants culture was performed as described previously [37, 38]. Briefly, after mice were sacrificed, left and right adrenal glands were collected and stored in ice‐cold DMEM/F12 (Cat. PM150312, Procell, China) supplemented with 0.1% bovine serum albumin (BSA; Cat. AR0004, Boster, China) until further processing. Each adrenal gland was cut into two halves (each containing both cortical and medullary tissue) and pre‐incubated in 200 µL of DMEM/F‐12 for 4 h at 37°C in 5% CO_2_ before treatment. After pre‐incubated, the culture medium was replaced with fresh medium containing the respective treatments, and explants were incubated for an additional 6 h at 37°C in 5% CO_2_. The treatment conditions were as follows: adrenal explants from WT mice were incubated with saline (control), or GnIH at 5.16 × 10^−11^ M (physiological concentration), 10^−9^ M (low pharmacological concentration), or 10^−7^ M (high pharmacological concentration), or GnIH (0.0516 nM) + RF9 (GPR147 antagonist, Cat. M03245, Beijing BioLab Technology Co., Ltd., China, 10^−6^ M); adrenal explants from GPR147 KO mice were incubated with saline (control), or GnIH (5.16 × 10^−11^ M). After the 6‐h stimulation, supernatants and explants were carefully removed and stored at −80°C until ELISA and gene expression analysis.

### 2.12 Retrograde Trans‐Synaptic PRV Tracing

PRV injection procedure was conducted with reference to the paper published by Xiaofeng Cheng [39]. We injected 3 µL of PRV‐EGFP virus (Cat. BC‐PRV‐803‐Plus, Brain Case Biotechnology Co., Ltd., China) into the left kidney of the mice. At 96 h post‐injection, to minimize nonspecific fluorescence from blood cells, mice were deeply anesthetized with 3% isoflurane and transcardially perfused with physiological saline (0.9% NaCl), followed by 4% paraformaldehyde. The brainstem and spinal cord were then harvested and post‐fixed in 4% paraformaldehyde.

### 2.13 Optogenetic Manipulation

Mice (n = 8 per group) were deeply anesthetized with 3% isoflurane and secured in a mouse stereotaxic frame (71001‐D, RWD Co., Ltd., China). Using bregma as the reference point, the intra‐LC injection coordinates were set as anteroposterior (AP) = −5.4 mm, mediolateral (ML) = ±1.0 mm, and dorsoventral (DV) = −3.8 mm. A total volume of 100 nL of AAV‐hSyn‐DIO‐hChR2(H134R) ‐EGFP (Cat. BC‐0627, Brain Case Biotechnology Co., Ltd., China) or aCSF was delivered at a controlled rate of 20 nL/min using a glass micropipette. Following injection, the pipette was left in place for 10 min to minimize backflow. Immediately after virus delivery, a fiber optic cannula was implanted into the same cranial window. Pre‐cleaned and polished fiber optic ferrules were positioned with the tip approximately 0.2 mm dorsal to the DV coordinate of the virus injection site to avoid physical compression of the core injection area.

Optogenetic manipulation was conducted 3 to 4 weeks after viral vector administration. During the experimental session, mice connected to optical fibers were placed in a behavioral testing apparatus and allowed to acclimate for at least 10 min. A predefined 8‐min light stimulation protocol was then initiated via the control software, consisting of alternating 30‐sec cycles of laser on and off. After treatments, serum was collected and stored at −80 °C, while the brainstem was fixed in neutral‐buffered formalin for histological analysis.

### 2.14 Statistical Analysis

All statistical analyses were performed using SPSS 22.0 (SPSS Inc., Chicago, IL, USA) and GraphPad Prism 10.2 (GraphPad Software, Inc., San Diego, CA, USA). Normality and homogeneity of variance were assessed using the Shapiro‐Wilk and Levene's tests, respectively. For two‐group comparisons, an unpaired two‐tailed Student's *t*‐test was used; for multiple‐group comparisons, one‐way ANOVA with Tukey's post‐hoc test was performed. Data are presented as mean ± SEM, with significance thresholds set at *p < 0.05 and **p < 0.01.

## 3 Results

### 3.1 GnIH and GPR147 Distributed in the LC‐SAM Axis of Mice

Immunofluorescence showed that GnIH‐ and GPR147‐immunoreactive (ir) neurons were distributed in the LC of the brainstem (Figure S1A) and the lateral horn of the thoracolumbar spinal cord (Figure S1B). Additionally, GPR147 immunoreactivity was observed in adrenal medullary chromaffin cells, while GnIH localized to a subset of ganglion cells and sympathetic nerve fibers within the adrenal medulla (Figure S1C). The negative control and antibody specificity validation were showed in Figure S7E. Furthermore, GnIH and GPR147 mRNA expression patterns in the LC and adrenal gland corroborated the immunofluorescence findings (Figure S1D).

### 3.2 Acute Stress Activated GnIH/GPR147 System of the LC‐SAM Axis in Mice

Three acute stress models‐restraint stress (RS), heat stress (HS), and lipopolysaccharide‐induced immune stress (LPS)‐were established to determine whether diverse acute stressors activate the GnIH/GPR147 system of the LC‐SAM axis. All stressors significantly increased circulating NE and EPI levels (*P* < 0.05 and *P* < 0.01, Figure 1B,C, Figure S2B,C), concomitant with elevated GnIH concentration (*P* < 0.05 and *P* < 0.01, Figure 1D, Figure S2D). However, corticosterone (CORT) levels remained unchanged across all models (Figure 1A, Figure S2A). In the LC, GnIH‐ and GPR147‐immunoreactive cell density increased markedly in all stress groups (*P* < 0.05 and *P* < 0.01; Figure 1E–G, Figure S2E‐‐G), but only LPS induced significant elevation of *GnIH* and *GPR147* mRNA expression (*P* < 0.05; Figure S2J). Additionally, tyrosine hydroxylase (TH; rate‐limiting enzyme for NE synthesis) expression was significantly upregulated in all stress groups (*P* < 0.05 and *P* < 0.01; Figure 1G,H, Figure S2H–K). In the adrenal glands, all stressors increased GPR147 immunoreactivity (*P* < 0.05; Figure 1I,J, S2L,N), but only RS significantly elevated GnIH immunoreactivity (*P* < 0.01; Figure 1I,J). The expression of TH and phenylethanolamine N‐methyltransferase (PNMT; converts NE to EPI) increased significantly, but *GnIH* and *GPR147* mRNA expression decreased dramatically across all stress groups (*P* < 0.05 and *P* < 0.01, Figure 1K,L, Figure S2O–R). To determine whether GnIH is released from LC terminals in an activity‐dependent manner during acute stress, we measured the concentrations of GnIH and NE following optogenetic activation of LC noradrenergic neurons. Our results showed that optogenetic activation of LC noradrenergic neurons significantly increased the number of TH‐positive and GnIH‐positive neurons in the LC, as well as the serum concentrations of GnIH and NE (Figure 1M–O).

**FIGURE 1:**
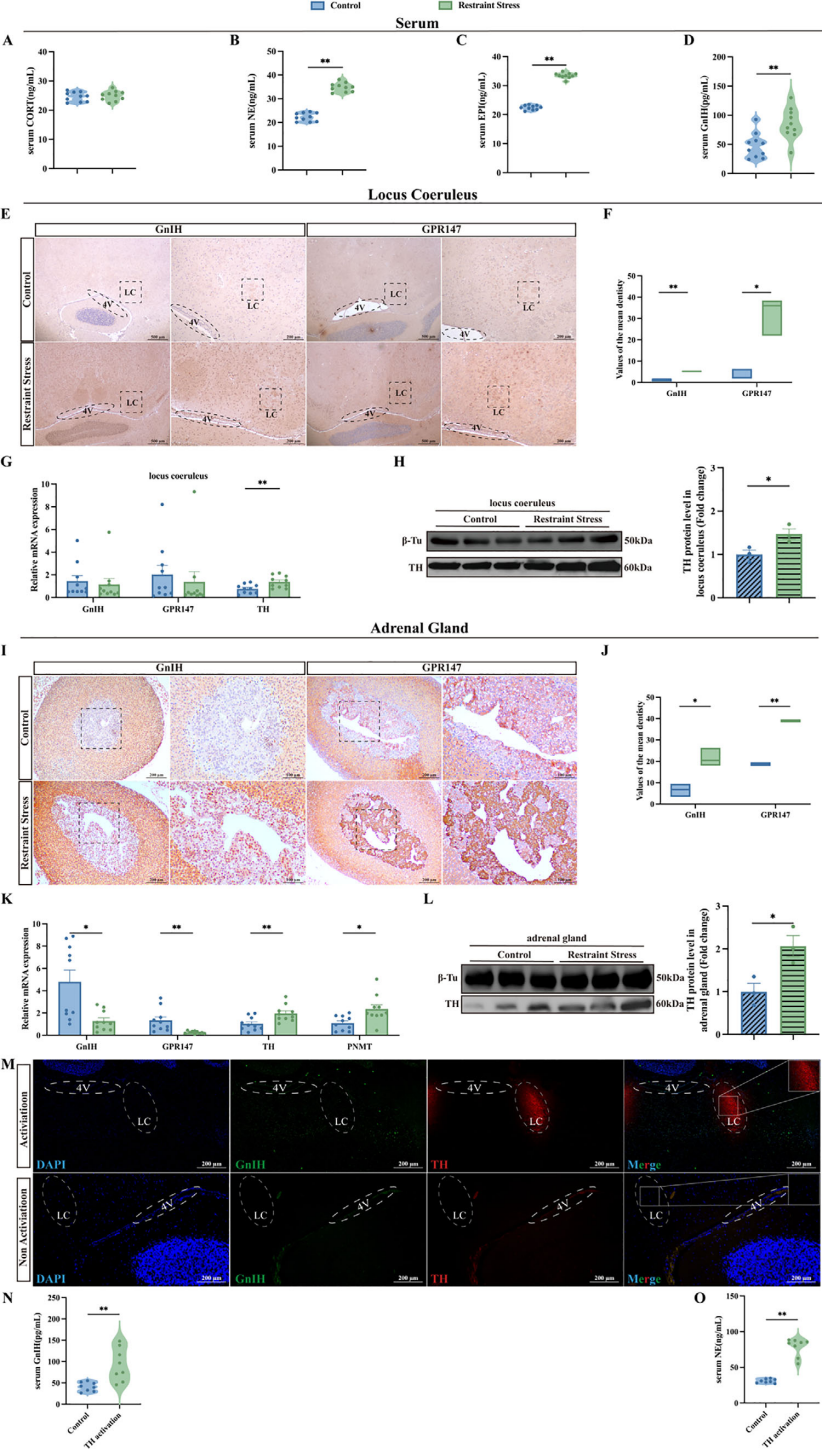
Activation of the GnIH/GPR147 System in the LC‐SAM Axis across Acute Restraint Stress Paradigms. (A to D): the concentration of CORT (A), NE (B), EPI (C), and GnIH (D) in the serum of mice under restraint stress (RS). (E and F): (E) The representative immunohistochemistry images and (F) mean densitometric values of GnIH or GPR147 in the locus coeruleus in acute restraint stress paradigms. (G and H): *GnIH*, *GPR147*, *TH* mRNA (G) and TH protein (H) levels in the locus coeruleus across acute restraint stress paradigms. (I and J): (I) Representative immunohistochemistry images and (J) mean densitometric values of GnIH or GPR147 in the adrenal gland in acute restraint stress paradigms. (K and L): *GnIH*, *GPR147*, *TH*, *PNMT* mRNA (K) and TH protein (L) in the locus coeruleus across diverse acute stress paradigms, including RS. (M) Optogenetic experiments demonstrated the expression of TH and GnIH neurons in the LC after the activation of norepinephrine neurons. Scale bar, 200 µm. (N and O) the concentration of GnIH (N) and NE (O) in the serum of mice after the activation of norepinephrine neurons. CORT: corticosterone; NE: norepinephrine; EPI: epinephrine; LC: locus coeruleus; TH: tyrosine hydroxylase; PNMT: phenylethanolamine N‐methyltransferase. GAPDH and β‐tubulin was used as an internal control. Data are mean ± SEM (n = 10 biological replicates per group). Comparisons between two groups were performed using two‐sided unpaired Student's *t*‐test with α = 0.05. For multiple comparisons, one‐way ANOVA followed by Tukey's post‐hoc test was used. Effect sizes (Cohen's d) were calculated for all significant comparisons. ^*^ *p* < 0.05, ^**^ *p* < 0.01, compared to the control group.

### 3.3 Acute Intraperitoneally Injected GnIH Induces Anxiety‐Like Behavior Increased and Neurotransmitter Metabolites Perturbations in Mice

To evaluate the effects of peripheral GnIH on stress‐related anxiety‐like behaviors and neuroendocrine profiles, we performed behavioral testing (open field test [OFT], elevated plus maze [EPM], and light dark box [LDB]) followed by targeted neurotransmitter metabolomics in mice receiving acute i.p. GnIH injections. Behavioral analyses revealed peripheral GnIH significantly elevated anxiety indices (*P* < 0.05; Figure 2G,P). This anxiogenic effect was manifested through avoidance of central zones (*P* < 0.05; Figure 2B,C,J), prolonged immobility duration (*P* < 0.05; Figure 2D,L), increased rearing (*P* < 0.05; Figure 2F,O) in the OFT and EPM, as well as increased time spent in dark box in the LDB and grooming frequencies in EPM (*P* < 0.05; Figure 2R,N). In addition, GnIH significantly decreased total locomotion (*P* < 0.05; Figure 2E,M) in both the OFT and EPM, reduced time spent in the open arms (EPM, *P* < 0.05; Figure 2I), and decreased time spent in the light box and the number of entries into the light box in the LDB (*P* < 0.05; Figure 2S–U).

**FIGURE 2:**
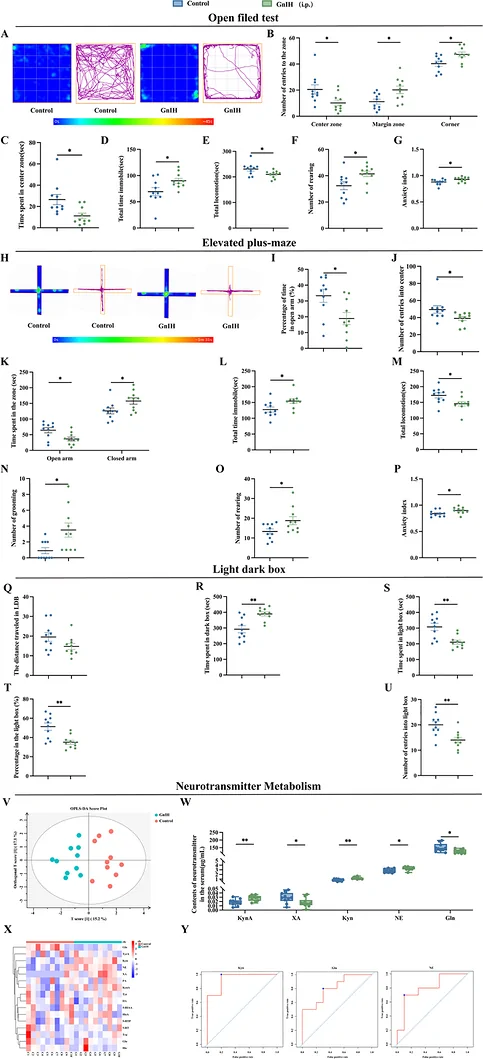
Effects of Acute Intraperitoneal GnIH Injection on Anxiety‐like Behaviors and Neurotransmitter Metabolomic Profiles in Mice. (A to G) Effects of acute intraperitoneal GnIH injection on anxiety‐like behaviors in mice during the open field test: (A) Motion track and heat map; (B) Number of entries to the zone; (C) Time spent in center zone; (D) Total time immobile; (E) Total locomotion; (F) Number of rearing; (G) Anxiety index. (H to P) Effects of acute intraperitoneal GnIH injection on anxiety‐like behaviors of mice during the elevated plus‐maze test: (H) Motion track and heat map; (I) Percentage of time in open arm; (J) Number of entries to the center; (K) Time spent in the zone; (L) Total time immobile; (M) Total locomotion; (N) Number of grooming; (O) Number of rearing; (P) Anxiety index. (Q to U) Effects of acute intraperitoneal GnIH injection on anxiety‐like behaviors of mice during the light dark box: (Q) The distance traveled in LDB; (R) Time spent in dark box; (S) Time spent in light box; (T) Percentage in the light box; (U) Number of entries into light box. (V) Orthogonal Projections to Latent Structures Discriminant Analysis (OPLS‐DA) score plots between GnIH and control groups. (W) The abundance of 5 significantly targeted metabolites in the metabolite of serum in the mice that received acute intraperitoneal GnIH injection. (X) Heat map of the 16 significantly targeted metabolites in the serum in the GnIH and control groups. The colors from red to blue indicate the relative contents of the metabolites in these two groups. (Y) The receiver operating characteristic (ROC) evaluates and screens potential biomarkers. The Area Under Curve (AUC) is to 1, the higher the accuracy of the prediction. Data are mean ± SEM (n = 10 biological replicates per group). Comparisons between two groups were performed using two‐sided unpaired Student's *t*‐test with α = 0.05. For multiple comparisons, one‐way ANOVA followed by Tukey's post‐hoc test was used. Effect sizes (Cohen's d) were calculated for all significant comparisons. ^*^ *p* < 0.05, ^**^ *p* < 0.01, compared to the control group.

Targeted metabolomic profiling of 24 serum neurotransmitters (Table 2) via mass spectrometry revealed distinct clustering patterns between GnIH‐treated and control groups in orthogonal partial least squares‐discriminant analysis (OPLS‐DA; Figure 2V). Of the 16 metabolites passing quality control thresholds, peripheral GnIH administration significantly reduced glutamine (Gln) and xanthurenic acid (XA; *P* < 0.05), but significantly elevated norepinephrine (NE), kynurenine (Kyn), and kynurenic acid (KynA) (*P* < 0.05‐0.01; Figure 2W). Receiver operating characteristic (ROC) analysis identified three biomarkers with robust diagnostic capacity for GnIH i.p injection: Gln (AUC = 0.81, sensitivity = 0.8, specificity = 0.7), Kyn (AUC = 0.96, sensitivity = 0.8, specificity = 1), and NE (AUC = 0.79, sensitivity = 0.7, specificity = 0.9) (Figure 2Y).

### 3.4 Acute Intraperitoneally Injected GnIH Induces the LC‐SAM Axis Activation in Mice

To investigate whether peripherally administered GnIH induces acute stress responses, we analyzed activation of the LC‐SAM axis and performed RNA‐sequencing of adrenal glands following acute i.p. GnIH injection in mice. Peripheral GnIH significantly elevated serum concentrations of CORT, NE, and EPI compared to vehicle controls (*P* < 0.05; Figure 3A). Notably, the concentration of GnIH in the cerebrospinal fluid (CSF) of control mice was higher than that in serum. However, acute i.p. GnIH injection significantly increased serum GnIH concentrations (*p* < 0.01; Figure 3B) but did not alter CSF GnIH levels, suggesting that peripherally administered GnIH does not cross the blood‐brain barrier. In the LC, i.p. GnIH injection markedly increased protein expression of TH, choline acetyltransferase (ChAT, definitive cholinergic neuron marker) and Norepinephrine transporter (NET) (*P* < 0.05‐0.01; Figure 3C,E). Transcriptional analysis revealed that GnIH obviously increased vesicular monoamine transporter 2 (*VMAT2*, critical for NE transport) mRNA levels, whereas significantly reduced *NET* mRNA expression (*P* < 0.05; Figure 3D). In the adrenal glands, i.p. GnIH injection significantly increased protein expression of TH and phenylethanolamine N‐methyltransferase (PNMT, rate‐limiting enzyme for NE synthesis) but markedly reduced NET protein levels (*P* < 0.05‐0.01; Figure 3F,J). Concurrently, i.p. GnIH injection significantly increased mRNA expression of *PNMT, VMAT 2* and *synaptophysin* (*P* < 0.05‐0.01; Figure 3G–I). In contrast, no significant differences in mRNA or protein levels of SNAP25 or syntaxin 1A were observed between the i.p. GnIH‐treated and control groups (Figure S4C,D). Principal component analysis confirmed high reproducibility (Figure S4E,F). RNA‐sequencing identified 128 DEGs in the adrenal glands following i.p. GnIH treatment, including 106 upregulated and 22 downregulated genes (Figure S4G). KEGG pathway enrichment analysis revealed that the DEGs were mainly enriched in the IL‐17 signaling pathway, MAPK signaling pathway, and NF‐κB signaling pathway (Figure 3K). Reactome annotation analysis indicated that most genes were annotated under signal transduction (Figure 3L). Gene set enrichment analysis (GSEA) demonstrated that the MAPK signaling pathway was positively correlated with GnIH treatment (Figure 3M). GO annotations revealed that DEGs were predominantly associated with stimulus response (Figure 3N). Among the DEGs, 34 were stress‐related. Analysis of the protein‐protein interaction (PPI) network topology of stress‐related DEGs showed that Fos proto‐oncogene (Fos), early growth response 1 (Egr1), and activating transcription factor 3 (Atf3) were the most important hub nodes (Figure 3O). qRT‐PCR validation confirmed GnIH‐induced upregulation of DNA damage‐inducible transcript 4 (*Ddit4*), salt‐inducible kinase 1 (*Sik1*), dual‐specificity phosphatase 1 *(Dusp1*), and insulin receptor substrate 2 (*Irs2*) (*P* < 0.01; Figure 3P).

**FIGURE 3:**
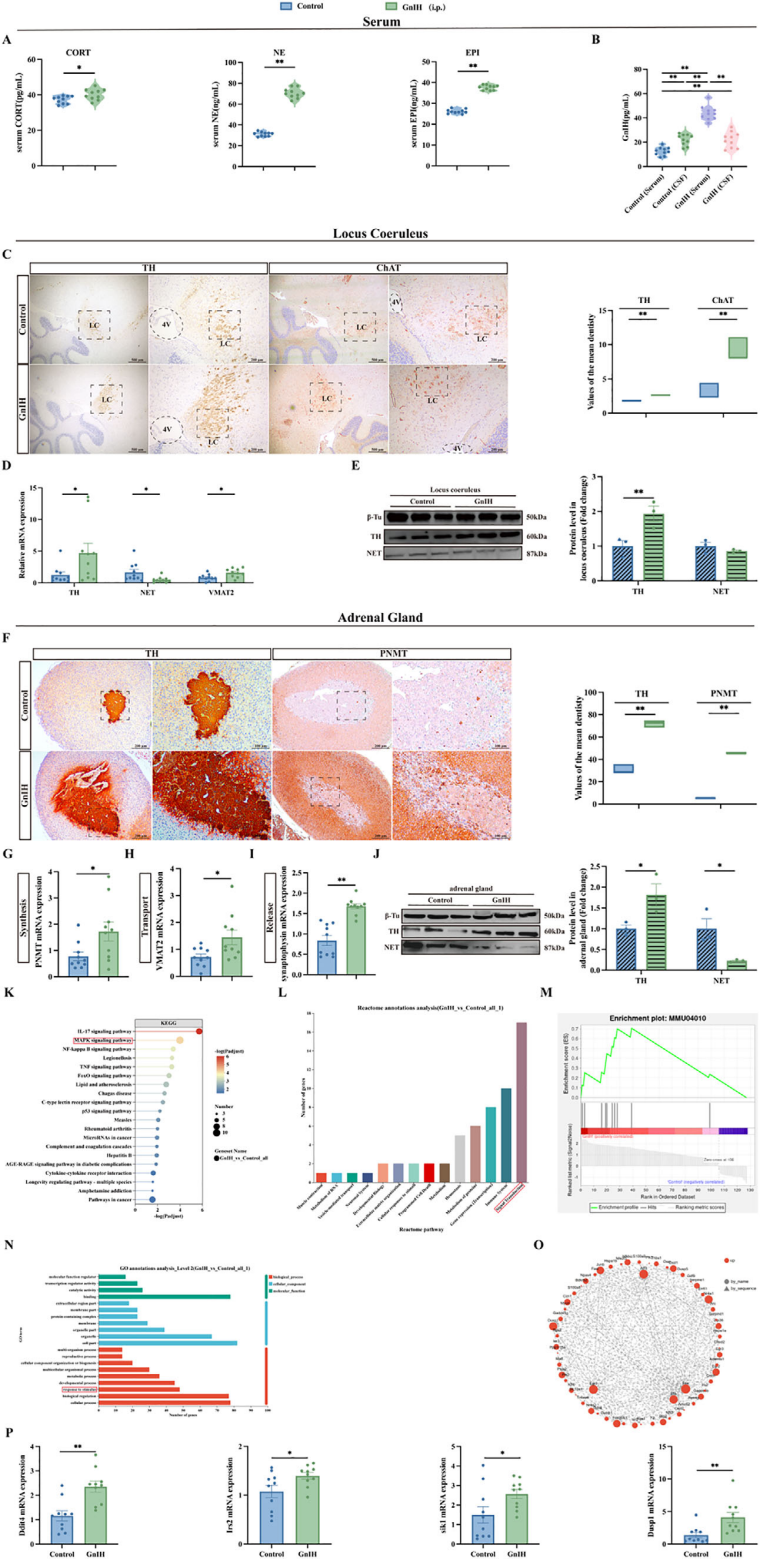
Effects of Acute Intraperitoneal GnIH Injection on LC‐SAM Axis Activation in Mice. (A and B) The concentrations of CORT, NE, EPI (A) and GnIH (B) in the serum or CSF of mice that received acute intraperitoneal GnIH injection. (C) Representative immunohistochemistry images and quantification of TH and ChAT immunoreactivity in the mouse locus coeruleus after acute intraperitoneal GnIH injection. Scale bar: 200/500 µm. (D and E): *TH*, *NET*, and *VMAT2* mRNA (D) and TH, NET protein (E) levels in the mouse locus coeruleus following acute intraperitoneal GnIH injection. (F) Representative immunohistochemistry images and quantification of TH and ChAT immunoreactivity in the mouse adrenal gland after acute intraperitoneal GnIH injection. Scale bar: 100/200 µm. (G to J): Adrenal mRNA (*PNMT*, *VMAT2*, and *synaptophysin*) and protein (TH and NET) levels after acute intraperitoneal GnIH injection. (K) Enriched KEGG pathways based on differential gene expression. (L) Reactome annotations analysis of differential gene expression. Areas of focus: signal transduction, immune system, gene expression (transcription), etc. (M) Adrenal gland treated with GnIH and vehicle were subjected to RNA‐seq and GSEA, showing enrichment of genes set related to MAPK signaling pathway. (N) GO classification of differentially expressed genes in the biological process (BP), cellular component (CC), and molecular function (MF), respectively. (O) PPI networks of DEGs related to stress. (P) Relative mRNA expression levels of *Ddit4*, *Irs2*, *Sik1*, and *Dusp1* as determined by real‐time quantitative PCR. Data are mean ± SEM (n = 10 biological replicates per group). Comparisons between two groups were performed using two‐sided unpaired Student's *t*‐test with α = 0.05. For multiple comparisons, one‐way ANOVA followed by Tukey's post‐hoc test was used. Effect sizes (Cohen's d) were calculated for all significant comparisons. ^*^ *p* < 0.05, ^**^ *p* < 0.01, compared to the control group.

### 3.5 GnIH or GPR147 Knockout Attenuates Acute Restraint Stress‐Induced Increase in Anxiety‐Like Behavior and LC‐SAM Axis Activation in Mice

To determine whether the GnIH/GPR147 system mediates acute stress‐induced increases in anxiety‐like behavior and LC‐SAM axis activation, we subjected GnIH or GPR147‐KO mice to acute restraint stress (RS) and assessed behavioral responses as well as markers of LC‐SAM axis activity. Genotyping of mutant mice was verified by PCR‐based analysis of genomic DNA. Validation results confirmed that GnIH and GPR147 were expressed in wild‐type (WT) mice but were absent in GnIH or GPR147 KO mice, indicating successful generation of the KO models (Figure S7A,B).

Compared with non‐stressed GnIH‐KO mice, RS‐treated GnIH‐KO mice showed no significant difference in the anxiety index measured in the OFT; however, the number of rearing events was significantly decreased (*p* < 0.01; Figure 4B,E). Furthermore, RS‐treated GnIH‐KO mice exhibited a significantly reduced anxiety index and decreased rearing frequency in the EPM (*p* < 0.05‐0.01; Figure 4G,M), as well as reduced time spent in the light box in the LDB test (*p* < 0.05; Figure 4N). Compared with RS‐treated WT mice, RS‐treated GnIH‐KO mice showed a significantly reduced number of grooming events, along with a decreased percentage of time spent in and entries into the closed arms, although the anxiety index in the EPM did not differ significantly (*p* < 0.05‐0.01; Figure 4H–M). Furthermore, RS‐treated GnIH‐KO mice exhibited significantly reduced time spent in the dark box and significantly increased time spent and percentage of time in the light box in the LDB test (*p* < 0.01; Figure 4N–P). Notably, serum concentrations of NE and EPI were significantly lower in GnIH‐KO mice than in WT mice under both RS and non‐stress conditions, whereas CORT levels were significantly higher (*P* < 0.01; Figure 4Q). In the LC, RS‐treated GnIH‐KO mice showed significantly upregulated of *VMAT2* mRNA expression compared to RS‐treated WT mice (*P* < 0.01; Figure 4R). In the adrenal glands, RS‐treated GnIH‐KO mice exhibited significantly reduced *TH*, *PNMT*, *NET*, *VMAT2* and *SNAP25* mRNA expression compared to RS‐treated WT mice, (*P* < 0.01; Figure 4S–U), while obviously decreased TH and synaptophysin protein levels (*P* < 0.05; Figure 4V). Furthermore, RS‐treated GnIH‐KO mice showed markedly decreased *synaptophysin* mRNA vs. non‐stressed GnIH‐KO mice (*P* < 0.05; Figure 4U).

**FIGURE 4:**
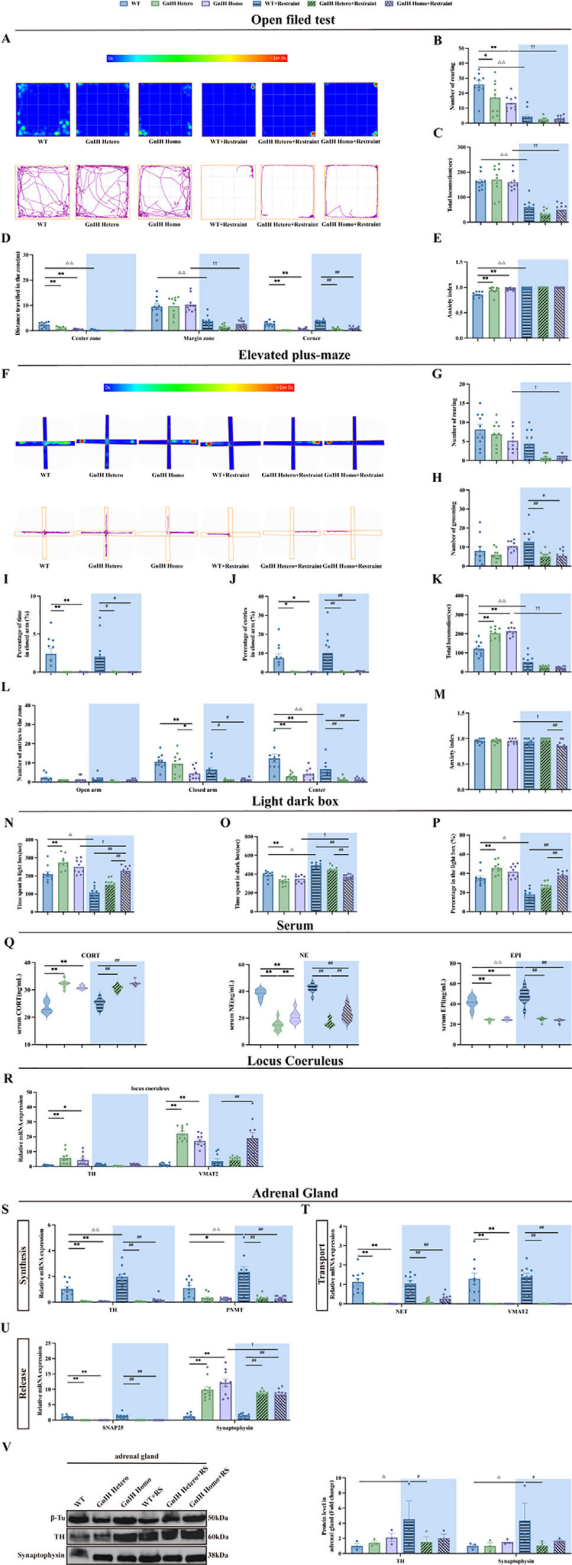
Effects of GnIH Knockout on Anxiety‐like Behaviors and LC‐SAM Axis Activation in Mice under Acute Restraint Stress. (A to E) Effects of GnIH knockout on anxiety‐like behaviors during the open field test following acute restraint stress: (A) Motion track and heat map; (B) Number of rearing; (C) Total locomotion; (D) Distance travelled in the zone; (E) Anxiety index. (F to M) Effects of GnIH knockout on anxiety‐like behaviors during the elevated plus‐maze test following acute restraint stress: (F) Motion track and heat map; (G) Number of rearing; (H) Number of grooming; (I) Percentage of time in closed arm; (J) Percentage of entries in closed arm; (K) Total locomotion; (L) Number of entries to the zone; (M) Anxiety index. (N to P) Effects of GnIH knockout on anxiety‐like behaviors during the light dark box following acute restraint stress: (N) Time spent in light box; (O) Time spent in dark box; (P) Percentage in the light box. (Q) Serum concentrations of CORT, NE and EPI in GnIH knockout mice following acute restraint stress. (R) *TH*, and *VMAT2* mRNA levels in the locus coeruleus of GnIH knockout mice following acute restraint stress. (S to V) Adrenal mRNA (*TH*, *PNMT*, *NET*, *VMAT2*, *SNAP25*, and *synaptophysin*) and protein (TH, and Synaptophysin) levels of GnIH knockout mice following acute restraint stress. GAPDH and β‐tubulin was used as an internal control. Data are mean ± SEM (n = 10 biological replicates per group). Comparisons between two groups were performed using two‐sided unpaired Student's *t*‐test with α = 0.05. For multiple comparisons, one‐way ANOVA followed by Tukey's post‐hoc test was used. Effect sizes (Cohen's d) were calculated for all significant comparisons. ^*^ *p* < 0.05, ^**^ *p* < 0.01, GnIH Hetero or GnIH Homo vs. WT. ^#^ *p* < 0.05, ^##^ *p* < 0.01, GnIH Hetero or GnIH Homo under RS vs. WT under RS. ^△^ *p* < 0.05, ^△△^ *p* < 0.01, WT vs. WT under RS. ^†^ *p* < 0.05, ^††^ *p* < 0.01, GnIH Homo vs. GnIH Homo under RS.

Notably, the behavioral phenotype of GPR147‐KO mice differed from that of GnIH‐KO mice. Although RS‐treated GPR147‐KO mice exhibited a significantly reduced number of rearing events and entries into the closed arms of EPM compared to non‐stressed GPR147‐KO mice (*p* < 0.05; Figure 5F,H), other behavioral parameters indicated that GPR147‐KO did not alleviate RS‐induced anxiety‐like behaviors. In contrast, serum concentrations of NE and EPI were significantly lower in GPR147‐KO mice than in WT mice under both RS and non‐stress conditions (*P* < 0.05‐0.01; Figure 5M), whereas CORT levels remained unchanged. In the LC, RS‐treated GPR147‐KO mice showed significantly decreased *TH* mRNA expression compared to RS‐treated WT mice (*P* < 0.01; Figure 5N). In the adrenal glands, RS‐treated GPR147‐KO mice showed significantly reduced expression of TH at both mRNA and protein levels, syntaxin 1A and synaptophysin mRNA levels, and SNAP25 protein levels relative to RS‐treated WT mice (Figure 5P–R). However, RS induced a significant upregulation of *PNMT* mRNA in GPR147‐KO mice compared to non‐stressed GPR147‐KO controls (Figure 5P).

**FIGURE 5:**
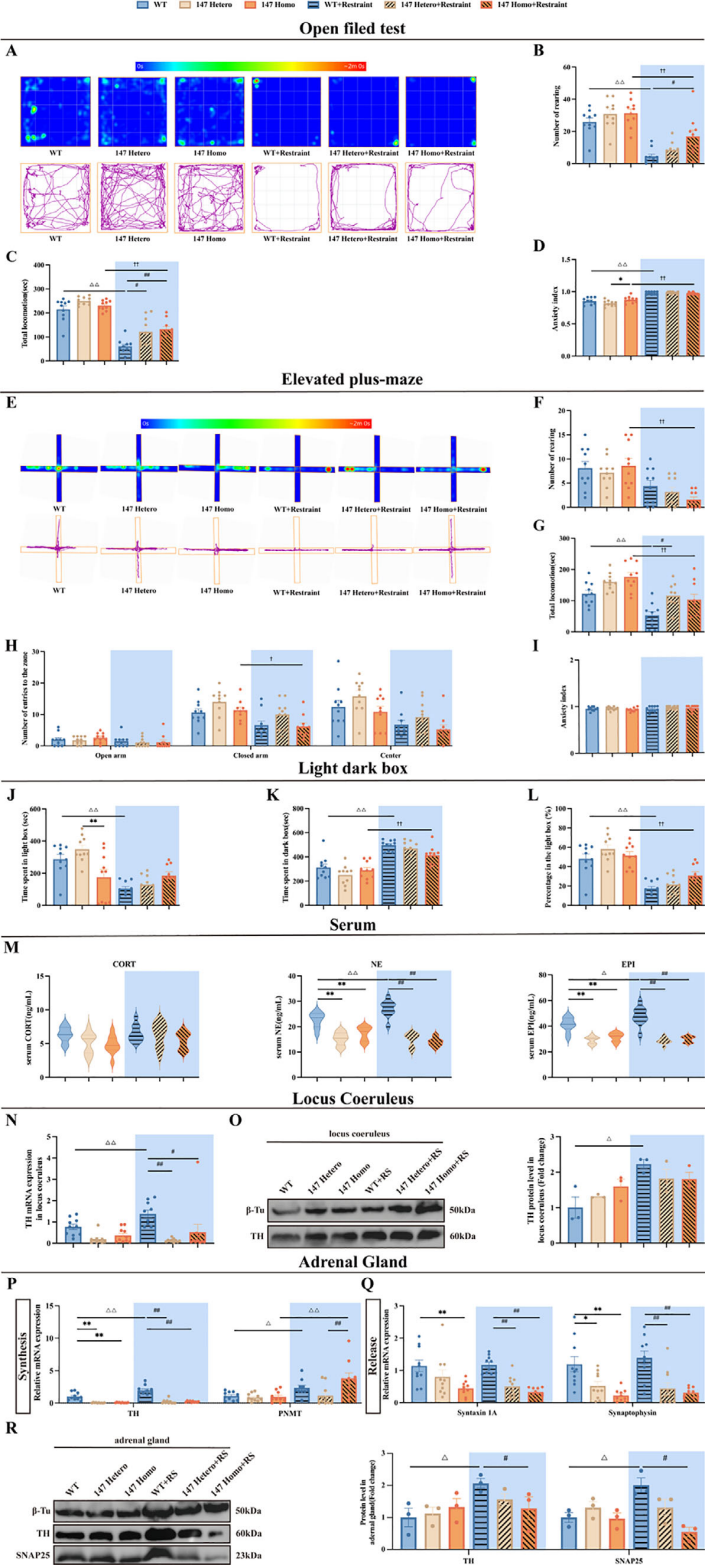
Effects of GPR147 Knockout on Anxiety‐like Behaviors and LC‐SAM Axis Activation in Mice under Acute Restraint Stress. (A to D) Effects of GPR147 knockout on anxiety‐like behaviors during the open field test following acute restraint stress: (A) Motion track and heat map; (B) Number of rearing; (C) Total locomotion; (D) Anxiety index. (E to I) Effects of GPR147 knockout on anxiety‐like behaviors during the elevated plus‐maze test following acute restraint stress: (E) Motion track and heat map; (F) Number of rearing; (G) Total locomotion; (H) Number of entries to the zone; (I) Anxiety index. (J to L) Effects of GPR147 knockout on anxiety‐like behaviors during the light dark box following acute restraint stress: (J) Time spent in light box; (K) Time spent in dark box; (L) Percentage in the light box. (M) Serum concentrations of CORT, NE and EPI in GPR147 knockout mice following acute restraint stress. (N and O) *TH* mRNA and TH protein levels in the locus coeruleus of GPR147 knockout mice following acute restraint stress. (P to R) Adrenal mRNA (*TH*, *PNMT*, *Syntaxin 1A* and *synaptophysin*) and protein (TH, and SNAP25) levels of GPR147 knockout mice following acute restraint stress. GAPDH and β‐tubulin was used as an internal control. Data are mean ± SEM (n = 10 biological replicates per group). Comparisons between two groups were performed using two‐sided unpaired Student's *t*‐test with α = 0.05. For multiple comparisons, one‐way ANOVA followed by Tukey's post‐hoc test was used. Effect sizes (Cohen's d) were calculated for all significant comparisons. ^*^ *p* < 0.05, ^**^ *p* < 0.01, GPR147 Hetero or GPR147 Homo vs. WT. ^#^ *p* < 0.05, ^##^ *p* < 0.01, GPR147 Hetero or GPR147 Homo under RS vs. WT under RS. ^△^ *p* < 0.05, ^△△^ *p* < 0.01, WT vs. WT under RS. ^†^ *p* < 0.05, ^††^ *p* < 0.01, GPR147 Homo vs. GPR147 Homo under RS.

### 3.6 GnIH Supplementation Enhances Norepinephrine Synthesis and Transport in Adrenal Explants In Vitro

To elucidate mechanisms underlying the direct effects of peripheral GnIH on adrenal NE biosynthesis, we performed immunofluorescence double‐staining assessing morphological relationship among GnIH/GPR147, and several sympathetic markers‐TH (a marker for catecholaminergic cells broadly), PNMT (a marker for adrenergic cells), and NPY (a marker for noradrenergic cells and some postganglionic fibers of the sympathetic nervous) in the adrenal glands, as well as in vitro assessments of NE synthesis, transport, and release in adrenal explants. Confocal microscopy revealed that GPR147 colocalized with TH, PNMT and NPY, whereas GnIH colocalized exclusively with PNMT and NPY in the adrenal medulla (Figure 6A). Electron microscopy showed that GnIH immunoreactivity was localized to or adjacent to norepinephrine‐containing granules in chromaffin cells of the adrenal medulla (Figure 6B). In the in vitro study, compared with the control group, GnIH at 5.16 × 10^−11^ M (physiological concentration) and 10^−9^ M significantly increased NE levels in the culture medium (*P* < 0.05‐0.01; Figure 7A), whereas no significant changes in CORT or EPI concentrations were observed across treatment groups. Furthermore, 5.16 × 10^−11^ M GnIH markedly upregulated the mRNA expression of NE synthesis‐related genes dopamine β‐hydroxylase (*Dbh*), and the NE transport‐related gene *NET* and *VMAT2* (*P* < 0.05‐0.01; Figure 7B,C), and also significantly increased NE release‐related genes *SNAP25* expression (*P* < 0.05; Figure 7D). However, no significant changes were observed in the mRNA levels of NE release‐related genes *syntaxin 1A* and *synaptophysin*. These results were validated by the finding that both pharmacological GPR147 antagonism (RF9 treatment) and genetic ablation of GPR147 significantly inhibited GnIH‐induced NE synthesis, transport and release, whereas no significant changes in CORT release were observed across treatment groups (*p* < 0.05‐0.01; Figure 7A–D).

**FIGURE 6:**
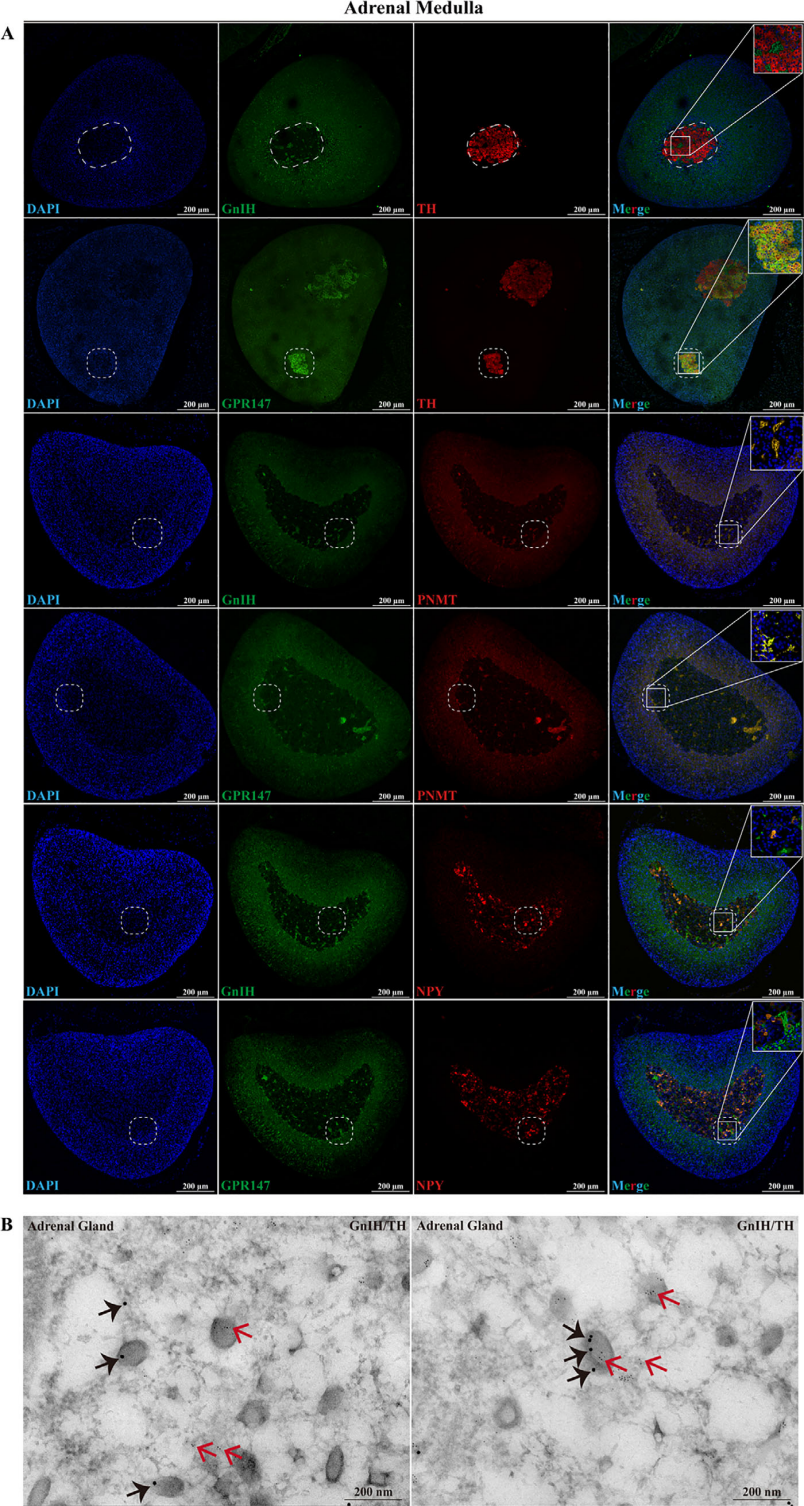
Dual immunofluorescence and double immunoelectron micrographs of GnIH/GPR147 system and TH, PNMT or NPY. (A) Colocalization of GnIH or GPR147 (green) and TH, PNMT or NPY (red) in the adrenal gland. Nuclear staining was performed with DAPI (blue). The yellow‐orange color indicates colocalization. (B) Double immunoelectron micrographs of the axons of GnIH and TH neurons in the adrenal gland. Black arrowheads show GnIH‐immunoreactive (ir) vesicles. Red arrowheads, TH‐ir vesicles. GnIH (12 nm colloidal gold) and TH (4 nm colloidal gold) localized on different neurosecretory vesicles. Scale bar, 200 µm.

**FIGURE 7:**
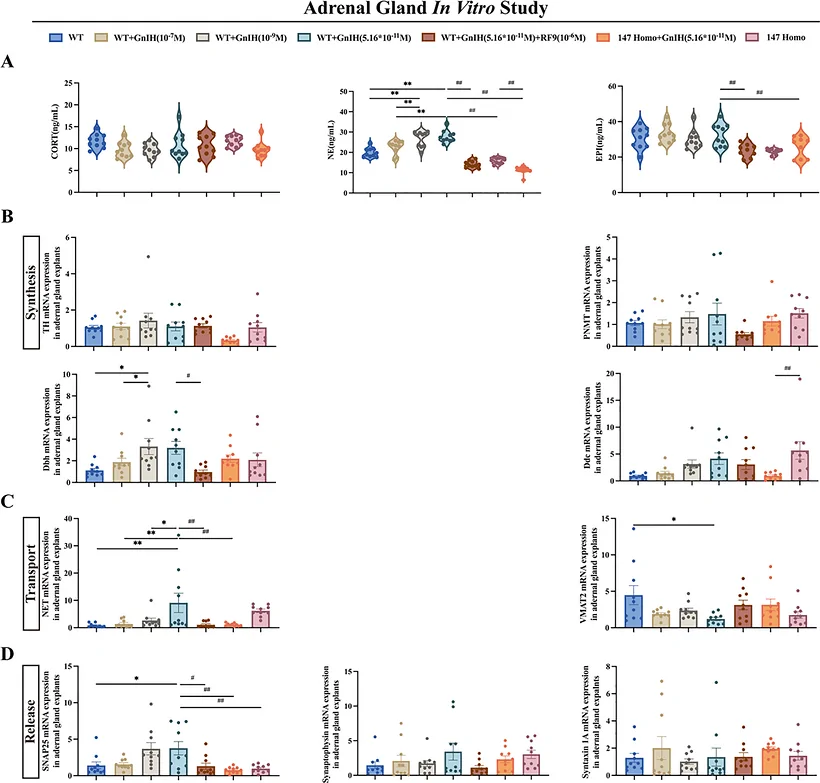
Effects of GnIH on Norepinephrine Synthesis, Transport and Release in Adrenal Explants. (A) Concentrations of CORT, NE, and EPI in the supernatant of adrenal explants of WT or GPR147 knockout treated with different doses of GnIH or RF9 for 6 h. (B‐D) Expression of catecholamine biosynthesis enzyme mRNAs (*TH*, *PNMT*, *Dbh* and *Ddc*), catecholamine transporter mRNAs (*NET* and *VMAT2*) and synaptic vesicle protein mRNAs (*SNAP25*, *Syntaxin 1A*, *Synaptophysin*) in adrenal explants of WT or GPR147 knockout treated with different doses of GnIH or RF9. Data are mean ± SEM (n = 10 biological replicates per group). Comparisons between two groups were performed using two‐sided unpaired Student's *t*‐test with α = 0.05. For multiple comparisons, one‐way ANOVA followed by Tukey's post‐hoc test was used. Effect sizes (Cohen's d) were calculated for all significant comparisons. ^*^ *p* < 0.05, ^**^ *p* < 0.01, between WT, WT plus GnIH (10^−7^M), WT plus GnIH (10^−9^M) and WT plus GnIH (5.16*10^−11^M). ^#^ *p* < 0.05, ^##^ *p* < 0.01, between WT plus GnIH (5.16*10^−11^M), WT plus GnIH (5.16*10^−11^M) plus RF9 (10^−6^M), GPR147 Homo plus GnIH (5.16*10^−11^M) and GPR147 Homo.

### 3.7 Acute Intracerebroventricular GnIH Injection Enhances Norepinephrine Synthesis, Transport and Release in LC‐SAM Axis

To elucidate mechanisms underlying central GnIH‐induced NE biosynthesis in the LC‐SAM axis, we characterized the morphological relationships between GnIH/GPR147 and TH (a catecholaminergic marker) or ChAT (a sympathetic preganglionic neuron marker), as well as the labeling of GnIH neurons retrogradely traced from the adrenal gland using PRV‐EGFP, in the LC and thoracolumbar spinal cord lateral horn. We also assessed NE synthesis, transport, and release following intracerebroventricular (i.c.v.) and intra‐LC administration of GnIH. Neuroanatomical localization revealed that GnIH‐ and GPR147‐immunoreactive (ir) neurons and fibers colocalized with TH‐positive neurons in the LC (Figure 8A). Notably, several GnIH‐ and GPR147‐ir neurons colocalized with ChAT‐ir neurons in the thoracolumbar spinal cord lateral horn (Figure 8D). At the electron microscopic level, a GnIH‐labeled axon terminal was observed to be localized adjacent to a TH‐labeled terminal in the LC (Figure 8C). Retrograde trans‐synaptic PRV tracing experiments showed that PRV‐infected neurons were mainly confined to the intermediolateral nucleus (IML) region of the lumbosacral spinal cord segments L3‐L5 (Figure 8E). In the LC, colocalization analysis demonstrated that several PRV‐infected neurons were GnIH‐positive (Figure 8B).

**FIGURE 8:**
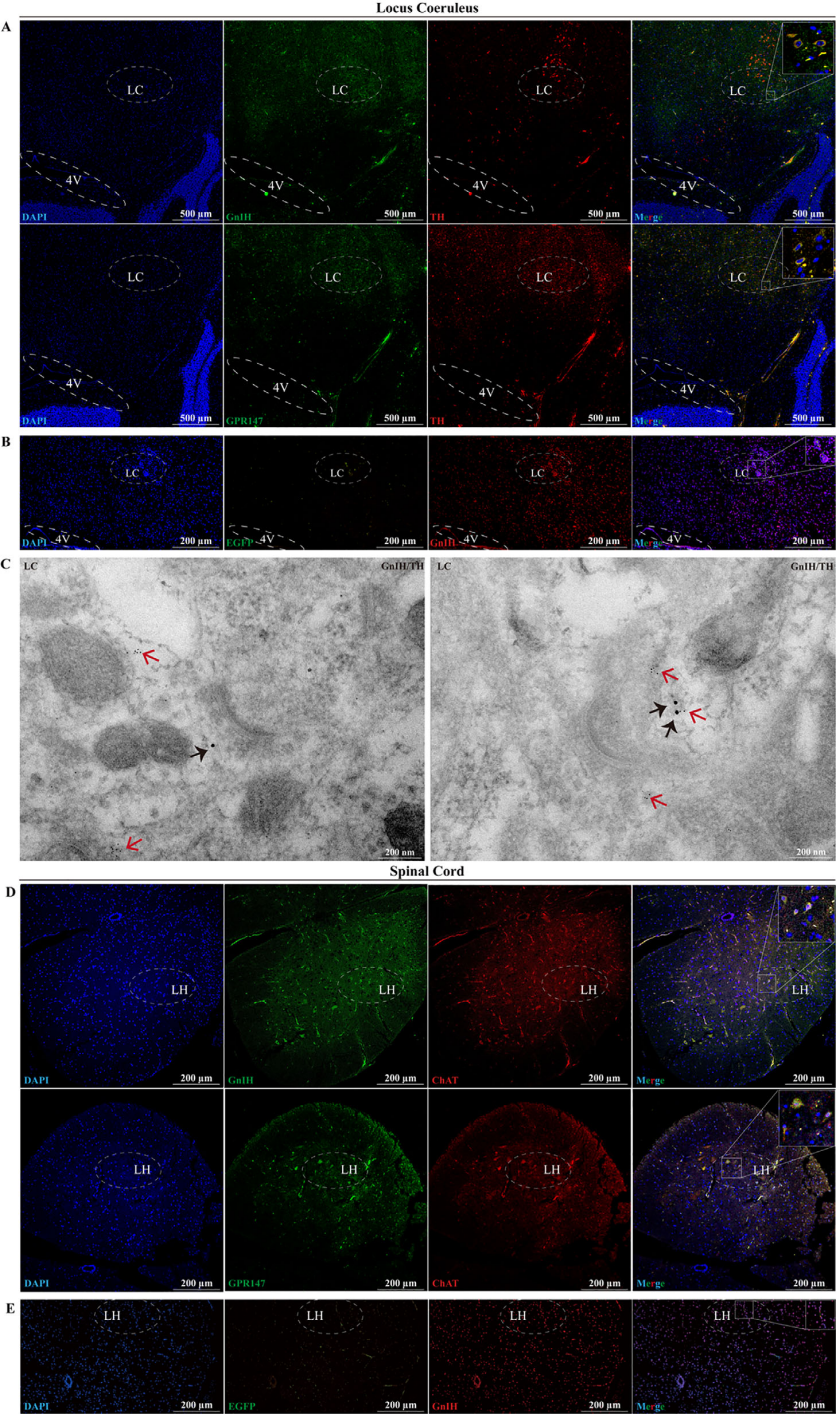
Dual immunofluorescence and double immunoelectron micrographs of GnIH/GPR147 system and TH or ChAT. (A) Colocalization of GnIH or GPR147 (green) and TH (red) in the locus coeruleus. Scale bar, 500 µm. (B) PRV‐infected GnIH neurons in LC. Scale bars, 200 µm. The yellow‐orange color indicates GnIH (red) and PRV‐EGFP (green) double‐positive neurons. Dashed lines delineate the outlines of the LC and the fourth ventricle. (C) Double immunoelectron micrographs of the axons of GnIH and TH neurons in the LC. Black arrowheads show GnIH‐ir vesicles. Red arrowheads show TH‐ir vesicles. GnIH (12 nm colloidal gold) and TH (4 nm colloidal gold) localized on different neurosecretory vesicles. Scale bar, 200 µm. (D) Colocalization of GnIH or GPR147 (green) and ChAT (red) in the spinal cord. Scale bar, 200 µm. Nuclear staining was performed with DAPI (blue). The yellow‐orange color indicates colocalization. (E) PRV‐infected GnIH neurons in spinal cord. Scale bars, 200 µm. The yellow‐orange color indicates GnIH (red) and PRV‐EGFP (green) double‐positive neurons. Scale bars, 200 µm.

Acute i.c.v injection of GnIH significantly increased serum concentrations of NE and EPI (*P* < 0.01; Figure 9A). In the LC, i.c.v GnIH injection significantly increased the protein expression of TH and ChAT (*P* < 0.05‐0.01; Figure 9B,D). Furthermore, i.c.v GnIH injection markedly increased *VMAT2* mRNA expression but significantly decreased *NET* expression mRNA levels (*P* < 0.05; Figure 9C). In the adrenal glands, i.c.v GnIH administration markedly upregulated the mRNA levels *PNMT*, *VMAT2*, *SNAP25*, *syntaxin 1A*, and the proto‐oncogene (*c‐Fos*), whereas it significantly downregulated *TH* and *NET* mRNA levels (*P* < 0.05‐0.01; Figure 9F–H). Moreover, i.c.v GnIH administration significantly increased the protein expression of PNMT, SNAP25 and syntaxin 1A (*P* < 0.05‐0.01; Figure 8E,I). Similar results were observed following intra‐LC GnIH administration. Intra‐LC GnIH injection significantly elevated serum EPI concentrations compared with vehicle controls (*p* < 0.05; Figure 9J). In the adrenal glands, intra‐LC injection significantly reduced *TH* mRNA expression (*p* < 0.05; Figure 9L) but markedly increased *SNAP25* mRNA levels (*p* < 0.01; Figure 9L).

**FIGURE 9:**
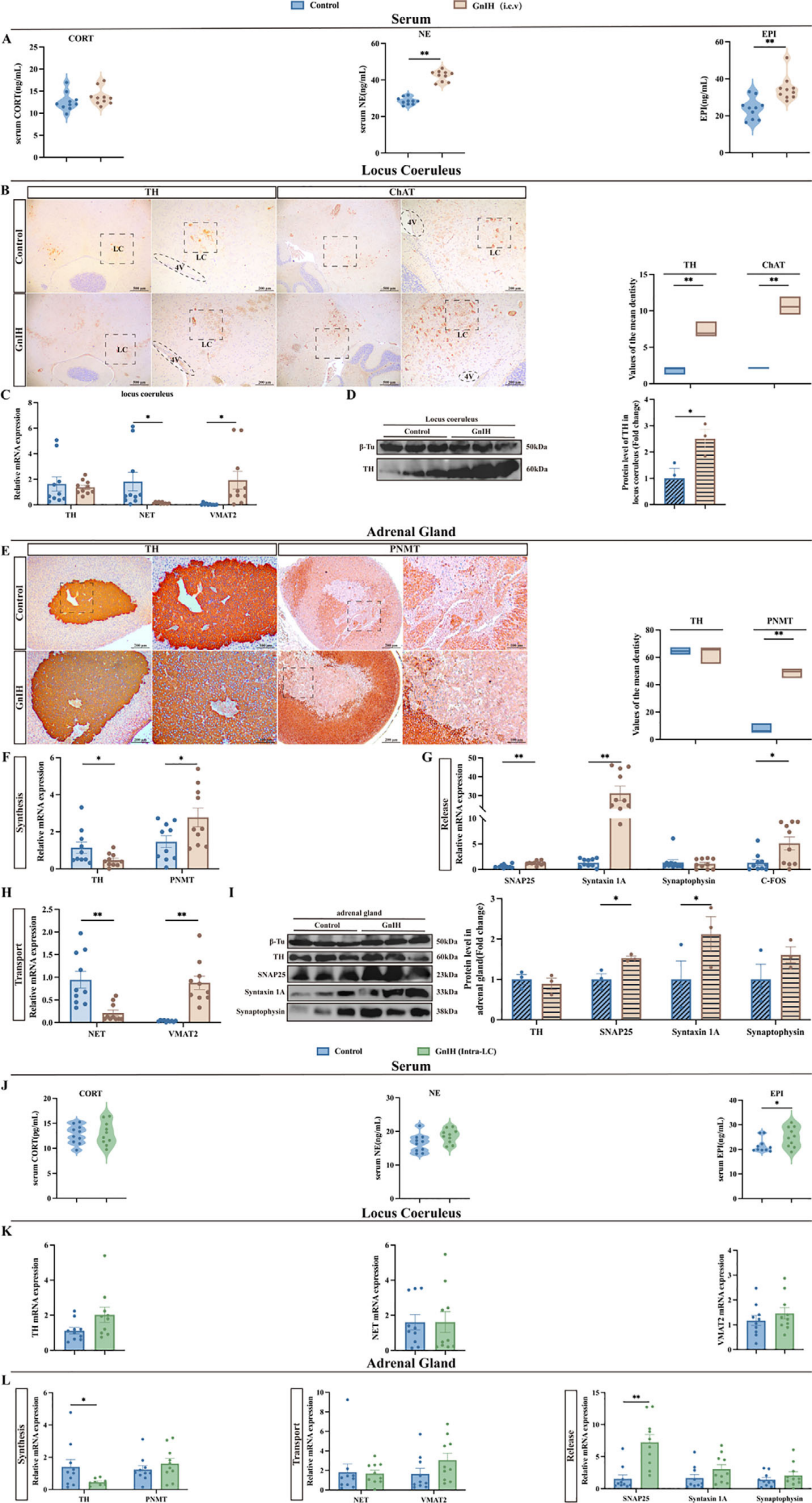
Effects of Acute Intracerebroventricular GnIH Injection on LC‐SAM Axis Activation and Norepinephrine Secretion in Mice. (A) Concentrations of CORT, NE, and EPI in serum following acute intracerebroventricular administration of GnIH. (B) Representative immunohistochemistry images and quantification of TH and ChAT immunoreactivity in the locus coeruleus after acute intracerebroventricular GnIH administration. Scale bar, 200/500 µm. (C and D): *TH*, *NET*, and *VMAT2* mRNA and TH protein levels in the locus coeruleus after acute intracerebroventricular GnIH administration. (E) Representative immunohistochemistry images and quantification of TH and PNMT immunoreactivity in the adrenal gland after acute intracerebroventricular GnIH administration. Scale bar, 100/200 µm. (F to I): Adrenal mRNA (*TH*, *PNMT*, *NET*, *VMAT2*, *SNAP25*, *Syntaxin 1A, synaptophysin*, and *C‐FOS*) and protein (TH, SNAP25, Syntaxin 1A, Synaptophysin) levels after acute intracerebroventricular GnIH administration. (J) Concentrations of CORT, NE, and EPI in serum following acute intra‐LC injection of GnIH. (K) *TH*, *NET*, and *VMAT2* mRNA levels in the LC after acute intra‐LC injection of GnIH. (L) Adrenal mRNA (*TH*, *PNMT*, *NET*, *VMAT2*, *SNAP25*, *Syntaxin 1A*, and *synaptophysin*) levels after acute intra‐LC injection of GnIH. LC: locus coeruleus; 4V: fourth ventricle; LH: spinal cord lateral horn. GAPDH and β‐tubulin was used as an internal control. Data are mean ± SEM (n = 10 biological replicates per group). Comparisons between two groups were performed using two‐sided unpaired Student's *t*‐test with α = 0.05. For multiple comparisons, one‐way ANOVA followed by Tukey's post‐hoc test was used. Effect sizes (Cohen's d) were calculated for all significant comparisons. ^*^ *p* < 0.05, ^**^ *p* < 0.01, compared to the control group.

### 3.8 Sympathetic Nerve Ablation Prevents Acute Intracerebroventricular Injected GnIH‐Induced Norepinephrine Elevation and LC‐SAM Axis Activation

To assess the contribution of autonomic ganglia and adrenal actions to GnIH‐induced NE elevation and LC‐SAM axis activation, we evaluated NE synthesis, transport, and release in mice subjected to sympathetic nerve ablation (SN abla) with i.c.v GnIH administration. In serum, concentrations of CORT and EPI showed no significant differences between the SN abla plus i.c.v GnIH group and either SN abla‐only or i.c.v GnIH‐only group. However, compared to i.c.v GnIH‐only group, the concentrations of NE significantly decreased (*P* < 0.01) in the SN abla plus i.c.v GnIH group and SN abla‐only group (Figure 10A). In the LC, compared to i.c.v GnIH‐only mice, the SN abla plus i.c.v GnIH group showed significantly decreased *TH* and *VMAT2* mRNA expression (*P* < 0.01), whereas significantly increased (*P* < 0.05) *NET* mRNA expression (*P* < 0.01; Figure 10B). In the adrenal glands, compared to i.c.v GnIH‐only group, the SN abla plus i.c.v GnIH group exhibited markedly reduced mRNA expression of *SNAP25* and *synaptophysin* as well as protein expression of SNAP25 (*p* < 0.05‐0.01; Figure 10F,G), while mRNA levels of *TH*, *PNMT*, *Dbh*, *Ddc*, *NET*, *VMAT2* and *Syntaxin 1A* showed no significant changes. Compared to the SN abla‐only group, the SN abla plus i.c.v GnIH group exhibited significantly reduced mRNA expression of *TH* and *VMAT2* (*P* < 0.05‐0.01; Figure 10D,E), while *PNMT*, *Dbh*, *Ddc*, *NET*, *SNAP25*, *synaptophysin* and *Syntaxin 1A* showed no significant changes. However, no differences in *TH*, *VMAT2*, or *NET* mRNA levels were detected between SN abla‐only and SN abla plus i.c.v GnIH groups.

**FIGURE 10:**
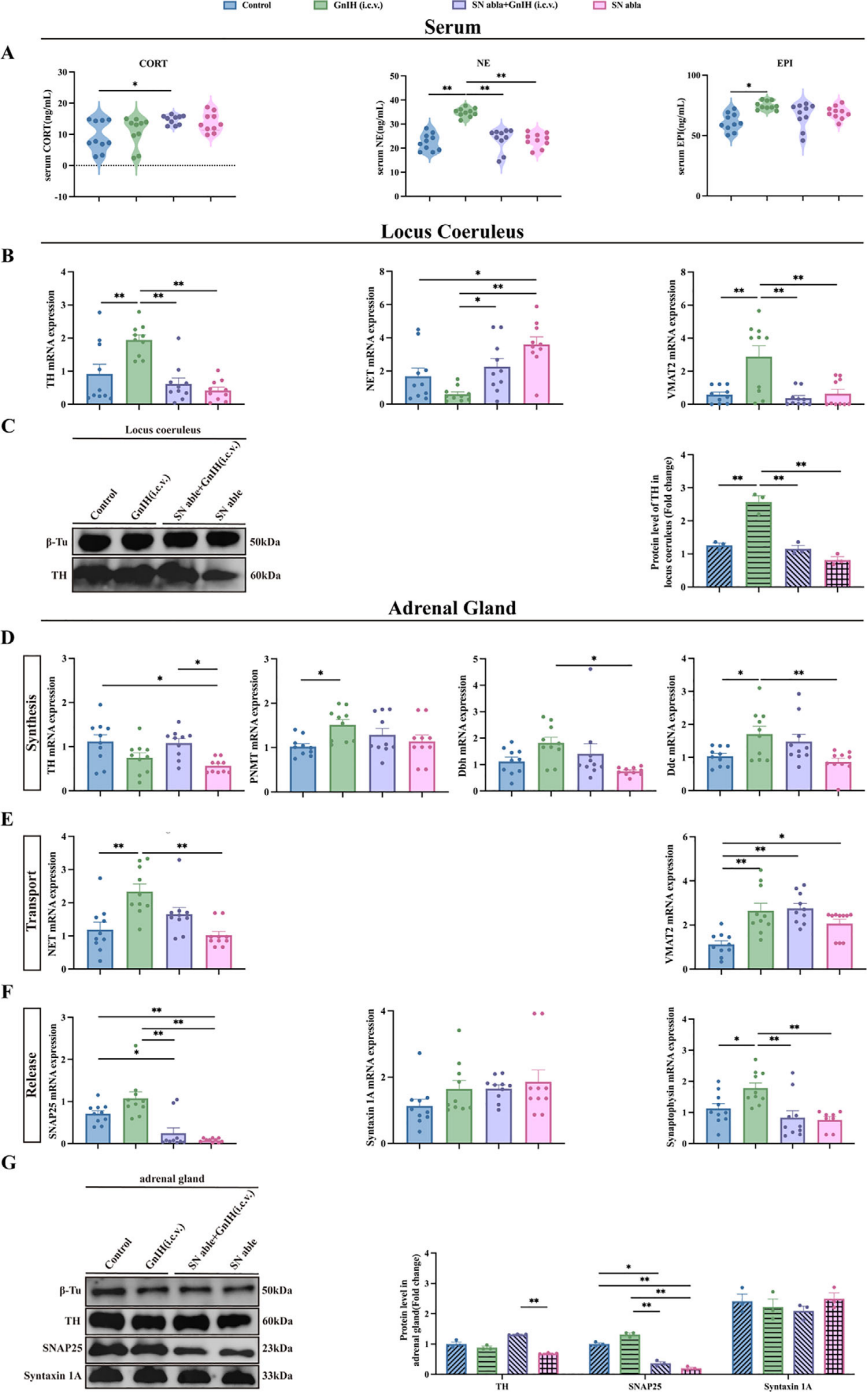
Effects of Acute Intracerebroventricular GnIH Injection on LC‐SAM Axis Activation and Norepinephrine Secretion in Mice with Sympathetic Nerve Ablation (A) Serum levels of CORT, NE, and EPI following acute intracerebroventricular GnIH administration in sympathectomized mice. (B and C) *TH*, *NET*, and *VMAT2* mRNA and TH protein levels in the locus coeruleus of sympathectomized mice after acute intracerebroventricular GnIH administration. (D to G) Expression of catecholamine biosynthesis enzyme mRNAs (*TH*, *PNMT*, *Dbh* and *Ddc*), catecholamine transporter mRNAs (*NET* and *VMAT2*), synaptic vesicle protein mRNAs (*SNAP25*, *Syntaxin 1A*, *Synaptophysin*) and protein (TH, SNAP25, Syntaxin 1A) levels in adrenal gland of sympathectomized mice after acute intracerebroventricular GnIH administration. GAPDH and β‐tubulin was used as an internal control. Data are mean ± SEM (n = 10 biological replicates per group). Comparisons between two groups were performed using two‐sided unpaired Student's *t*‐test with α = 0.05. For multiple comparisons, one‐way ANOVA followed by Tukey's post‐hoc test was used. Effect sizes (Cohen's d) were calculated for all significant comparisons. ^*^ *p* < 0.05, ^**^ *p* < 0.01, between the indicated groups.

## 4 Discussion

Substantial evidence demonstrates that chronic or maladaptive stress serves as a primary etiological factor in psychiatric disorders and systemic pathologies [40, 41]. Acute stress constitutes the fundamental biological basis for these effects, yet the neuroendocrine mechanisms governing sympathetic activation during acute stress remain incompletely characterized. Given the established role of the GnIH/GPR147 system as a critical neuroendocrine stress regulator in mammals, this study elucidates the functional relationship between GnIH/GPR147 signaling and acute stress responses, with specific focus on its mechanistic control of LC‐SAM axis activation.

Since its initial identification in the avian hypothalamus [42], accumulating morphological evidence indicates that GnIH participates in diverse physiological functions beyond its established reproductive roles, particularly in stress‐reproduction interactions. Immunohistochemistry (ICC) studies have demonstrated that GnIH neuronal cell bodies are located in the paraventricular nucleus (PVN) in birds and the dorsomedial hypothalamic area (DMH) in most mammals [16, 43]. Furthermore, GnIH‐ir fibers and receptors exhibit widespread distribution throughout the limbic system, extending caudally to the brainstem and potentially the spinal cord [44]. Confocal microscopy combined with double‐label immunohistochemistry subsequently revealed colocalization of glucocorticoid receptors (GR), corticotropin‐releasing hormone (CRH), and α2A‐adrenergic receptors within PVN GnIH neurons [14, 45]. Despite accumulated studies delineating the precise localization of GnIH within the hypothalamus across various species, its extrahypothalamic and adrenal localization patterns remain poorly defined. Previous investigations confined GnIH‐ir fibers to the brainstem's medial longitudinal fasciculus and spinal cord's dorsal horn [46]. Our study significantly advances neuroanatomical understanding by demonstrating GnIH‐ir and GPR147‐ir neuronal distributions in both the locus coeruleus (LC) and the lateral horn of the thoracolumbar spinal cord. Accumulated evidence has verified that the GnIH receptor GPR147 is distributed in numerous peripheral organs and tissues. In addition to reproductive organs, our previous studies and those of others have revealed GnIH and GPR147 expression in adipose tissue, pancreas, gastrointestinal tract, and liver [47, 48]. In the present study, we observed that GnIH‐ir is primarily localized to ganglion cells and sympathetic nerve fibers, whereas GPR147‐ir is predominantly found in chromaffin cells within the adrenal medulla. This work provides the first comprehensive anatomical evidence of GnIH/GPR147 system throughout the LC‐SAM axis, suggesting its potential regulatory role in acute stress responses‐acting not only through the central nervous system but also directly modulating the adrenal gland.

In 2008, Calisi et al. first demonstrated that acute capture handling stress significantly increased hypothalamic GnIH‐ir neurons in house sparrows, representing the inaugural report of stress‐induced GnIH system modulation in avian species [49]. Subsequent research has established the diverse stressors and their duration, such as physical or psychological, and acute or chronic, can modulate the GnIH system. Kirby et al. documented increased GnIH mRNA and peptide expression in the DMH of male rats following both acute and chronic restraint stress [11]. Complementary murine studies further revealed rapid activation of GnIH neurons during acute restraint stress. Additional investigations demonstrated that metabolic stress (acute fasting) [50], environmental stress (heat exposure) [51] and nociceptive stress (electric foot‐shock) [52] significantly upregulated hypothalamic GnIH expression and neuronal activity in avian and murine models. Beyond physical stressors, psychological challenges (acute social defeat) and pharmacological stressors (LPS/CORT administration) similarly induced hypothalamic GnIH neuronal activation in Nile Tilapia and testicular GnIH upregulation in European Starlings [53]. Our findings align with this established paradigm, demonstrating that acute restraint, thermal, and immune stressors consistently elevated serum GnIH concentrations and activated GnIH/GPR147 signaling in LC and adrenal gland, indicating evolutionarily conserved GnIH/GPR147 system activation by acute stressors from fish to mammals. Physiologically, stress responses involve rapid phase: LC‐SAM axis activation triggering catecholamine release (EPI/NE) and delayed phase: HPA axis activation triggering glucocorticoid secretion (CORT/cortisol) [54]. Notably, our data revealed that acute stress induced concurrent serum GnIH elevation and increased NE/EPI release across models, while CORT levels remained unchanged. Given that acute restraint typically elevates CORT levels in mice, this discrepancy is noteworthy. We speculate that it may reflect differences in basal CORT levels, housing conditions, or the intensity of the stress stimulus, all of which can modulate the magnitude of the HPA axis response. Concurrently, key noradrenergic (TH, PNMT) and GnIH system components (GnIH/GPR147) were upregulated in LC and adrenal tissues. These results demonstrate that acute stressors not only activates the LC‐SAM axis but also the GnIH system components in the LC‐SAM axis, indicating a critical role for GnIH system components in acute stress responses. By definition, NE co‐transmitters are substances that are co‐released with NE during sympathetic activation to potentiate or synergize NE effects [9]. Existing literature indicates that NE release from sympathetic nerve terminals is generally Ca^2^^+^‐dependent [55]. However, the Ca^2^^+^ dependency and frequency sensitivity of GnIH‐induced NE real‐time release have not been directly examined. We speculate that GnIH, as a potential NE co‐transmitter, may influence frequency‐dependent NE release by modulating N‐type calcium channels, but this possibility warrants further investigation.

Although substantial evidence confirms acute stressors activate the GnIH/GPR147 system, the functional consequences of GnIH signaling on stress responses remain poorly characterized. During stress adaptation, organisms modulate behavioral and neuroendocrine outputs to restore homeostasis [56]. We therefore investigated GnIH effects on anxiety‐like behaviors and neuroendocrine profiles in mice following acute i.p GnIH administration. Behavioral analyses revealed peripheral GnIH significantly elevated anxiety indices, manifested through avoidance of central zones in OFT and EPM, prolonged immobility duration, and increased rearing and grooming frequencies. Complementary rat studies demonstrated that i.c.v. GnIH similarly induces anxiety phenotypes in open‐field tests [25]. These findings establish that both central and peripheral GnIH administration promote anxiety‐related behaviors. Notably, acute i.p GnIH treatment in mice elevated serum NE, Kyn, and KynA, while reduced Gln and XA concentrations. NE represents the canonical LC‐SAM axis activation marker [57]. Gln depletion disrupts glutamate‐glutamine cycle equilibrium, contributing to stress pathogenesis [58]. Furthermore, Kyn metabolism bifurcates into: neuroprotective KA pathway and neurotoxic branch producing KynA [59]. Increased KynA triggers stress responses that may precipitate neuropsychiatric disorders including anxiety and depression [60]. Our results demonstrate that acute peripheral GnIH administration induces stress responses through neurotransmitter profile alterations, mechanistically linking GnIH signaling to neurometabolic stress pathways.

To investigated the mechanism by which acute peripheral administered GnIH induces acute stress responses, we analyzed LC‐SAM axis activation and performed adrenal gland RNA‐sequencing in mice following acute i.p GnIH injection found that i.c.v administration of GnIH increases the plasma ACTH levels in rats [52]. Subsequently, reported that i.c.v. injections of GnIH elevated CORT release in mice [20]. Our previous study consistent with these results that chronic i.p. injected GnIH significantly increased the concentrations of CORT and ACTH in chicken [32]. Notably, our present study verified that acute i.p. GnIH administration obviously elevated not only CORT levels but also NE and EPI concentration. Despite this being the first report to reveal GnIH could induced NE and EPI release, previous study shown that NE treatment increases GnIH release from diencephalic tissue blocks in vitro and that GnIH neurons express α2A‐adrenergic receptor [14], suggesting bidirectional interaction between GnIH and NE, as well as CORT. It is well known that NE serves as a hallmark neurotransmitter of acute stress responses, which 70% is synthesized and secreted by post‐synaptic sympathetic neurons and noradrenergic neurons in the brain, while the remaining 30% is synthesized and secreted by the adrenal medulla chromaffin cells [61]. During acute stress, the LC and adrenal medulla enhance the synthesis of rate‐limiting enzymes for NE production, including TH and PNMT [62]. Following their synthesis in adrenal medullary chromaffin cells, NE and EPI are released through vesicle‐plasma membrane fusion mediated by SNARE protein complexes (e.g., Syntaxin 1A, SNAP25, VAMP2) and regulatory proteins such as synaptophysin [63]. Post‐exocytosis, cellular CAs homeostasis is maintained through NET‐mediated presynaptic reuptake and vesicular storage driven by VMAT2 [64]. Additionally, ChAT, a classical marker of cholinergic neurons, may exhibit upregulated expression in sympathetic neurons under stress conditions [65]. Our results demonstrated that GnIH significantly increased the expression of TH, PNMT, VMAT2 and synaptophysin in both the LC and adrenal glands, suggesting that peripheral GnIH enhances NE synthesis, transport and release. Furthermore, our adrenal RNA‐sequencing results identified 34 stress‐related differentially expressed genes (DEGs) following GnIH treatment, which were mainly enriched in the MAPK signaling pathway. Studies have reported that MAPK signaling pathway activation is positively correlated with biological responses to NE [66, 67]. Thus, the adrenal RNA‐sequencing data further confirm that peripheral GnIH triggers activation of neuroendocrine stress‐response pathways.

Based on the above findings, we hypothesized that GnIH mediates acute stress‐induced LC‐SAM axis activation and NE secretion. To validate this hypothesis, GnIH‐KO and GPR147‐KO mice were utilized to evaluate anxiety‐like behaviors and LC‐SAM axis activation under acute restraint stress. Behavioral tests revealed that both GnIH‐KO and GPR147‐KO mice exhibited significantly attenuated anxiety‐like behaviors when subjected to acute restraint stress. These results are consistent with previous studies demonstrating that GnIH‐KO mice display reduced anxiety levels, and that central administration of GnIH to GnIH‐KO mice restores anxiety‐like responses in the elevated plus maze test [25]. Furthermore, i.c.v. injection of RF9, a GPR147 antagonist, was shown to significantly reverse anxiety‐like effects induced by amphetamine withdrawal [68]. Notably, our present study further revealed that serum concentrations of NE and EPI were significantly lower in both GnIH‐KO and GPR147‐KO mice compared to wild‐type controls, under both acute restraint stress and non‐stress conditions. Additionally, NE synthesis and transport in the LC, as well as NE synthesis, transport, and release in the adrenal glands, were markedly reduced in acutely stressed GnIH‐KO mice relative to wild‐type mice. Collectively, our findings, together with previous evidence, demonstrate that the GnIH/GPR147 system plays a critical role in mediating acute stress‐induced LC‐SAM axis activation and NE secretion.

NE is synthesized by noradrenergic neurons in the LC and by chromaffin cells in the adrenal medulla, from which it is released. Approximately 70% of NE is released from sympathetic nerve endings into the capillaries, while the remaining portion is secreted by chromaffin cells into the bloodstream [8, 69]. Although accumulated evidence indicates that GnIH‐ir neurons are clustered in the mediobasal hypothalamus and exhibit extensive projections and terminals throughout the central nervous system (CNS) [44], it remained unknown whether the GnIH/GPR147 system projects to noradrenergic neurons or chromaffin cells. Our neuroanatomical study demonstrates, for the first time, that GnIH and GPR147 fibers contact noradrenergic neurons in the LC. Additionally, sympathetic neurons in the thoracolumbar spinal cord lateral horn were found to express both GnIH and GPR147. These findings suggest that the GnIH/GPR147 system may directly regulate central NE synthesis and sympathetic nervous activation within the CNS. It is worth noting that chromaffin cells in the adrenal medulla also express the GnIH/GPR147 system, and GnIH was observed to colocalize with NPY. Given that NPY is currently recognized as a marker of sympathetic neurotransmission [70], our anatomical evidence indicates that GnIH may not only directly regulate adrenal NE synthesis in an autocrine/paracrine manner but also may function as a novel sympathetic neurotransmitter. To further investigate the central and peripheral mechanisms through which GnIH/GPR147 mediates acute stress regulation, we evaluated the activation of the LC‐SAM axis and NE secretion following i.c.v administration of GnIH in mice and in vitro cultured adrenal explants. Our in vitro findings demonstrate that GnIH, at both physiological and pharmacological concentrations, significantly enhances the concentration of NE in the culture supernatant, as well as the synthesis, transport and release of NE in adrenal explants. These results were validated by the finding that both pharmacological GPR147 antagonism (RF9 treatment) and genetic ablation of GPR147 significantly inhibited GnIH‐induced NE synthesis, transport and release, whereas no significant changes in CORT release were observed across treatment groups. Interestingly, i.c.v GnIH treatment not only markedly increased NE synthesis and transport in both the LC and adrenal gland but also elevated NE release from the adrenal gland. These results were corroborated by sympathectomy combined with i.c.v GnIH administration: sympathetic ablation blocked GnIH‐induced NE synthesis and transport in the LC, as well as NE release from the sympathetic nerve endings in the adrenal medulla, but did not affect adrenal NE synthesis and transport alone. These findings suggest that the GnIH/GPR147 system can directly regulate LC‐SAM axis activation and NE secretion through dual central and peripheral pathways. Specifically, GnIH may act directly on chromaffin cells to increase adrenal NE synthesis and secretion via an autocrine/paracrine mechanism within the adrenal medulla. Simultaneously, GnIH may also directly regulate noradrenergic neurons to enhance NE synthesis, thereby acting as a co‐transmitter with NE to activate and promote sympathetic nerve‐mediated NE release. However, given that GnIH‐ir neurons are clustered in the mediobasal hypothalamus, the projection patterns and neural circuit mechanisms connecting the hypothalamus to the LC also require further investigation using advanced neurobiological techniques.

In summary, our data show that the GnIH/GPR147 system mediates acute stress‐induced activation of the LC‐SAM axis and an increase in anxiety‐like behaviors, indicating a role for GnIH in the acute stress response. Furthermore, the present findings reveal a function of GnIH mimics or augments endogenous NE signaling to activate the LC‐SAM axis during acute stress. This research provides a neuroendocrine perspective on the mechanistic understanding of acute stress regulation. Pharmacological modulation of the GnIH/GPR147 system may offer a therapeutic approach for inhibiting maladaptive stress responses and stress‐related pathologies and infertility.

## Author Contributions

Jiani Zhang conducted the experiments, wrote the primary manuscript, and performed statistical tests. Wenqi Wang and Hongyu Zhu conducted the experiments and performed statistical tests. Bingqian Shen, Yuanyuan Xin, Xingxing Song, Xuanyi Lu, and Lixuan Li conducted animal feeding and care. Siting Chen, Xiaochen Zhang, Jialing Zeng, and Haodong Zheng collected sam ples. Jianlong Lao, and Ke Peng helped the animal experiments. Yixian Wei, Jiaming Zheng, Xiaoye Wang, and Chuanhuo Hu edited and revised the manuscript. Xun Li conceived and designed the project. All authors read and approved the final manuscript.

## Funding

This work was supported by the National Natural Science Foundation of China (NSFC) (32160818,32360864), the Natural Science Foundation of Guangxi Province (2021GXNSFAA196001) and the Innovation Project of Guangxi Graduate Education (YCSW2024126).

## Conflicts of Interest

The authors declare no conflicts of interest.

## Ethics Statement

All of the experiments were performed in accordance with the guidelines of the Regional Animal Ethics Committee and were approved by the Guangxi University Ethical Committee, approval number (GXU‐2025‐0104).

## Supporting information




**Supporting File**: advs78176‐sup‐0001‐SuppMat.xlsx.

## Data Availability

All data generated or analyzed during this study are included in this article. Further inquiries can be directed to the corresponding author.
